# Explainable Two-Stage Xception-Swin Transformer Learning for Body-Part-Aware Fracture Detection in Musculoskeletal X-Rays

**DOI:** 10.3390/jimaging12070298

**Published:** 2026-07-03

**Authors:** Syed Baqir Hussain Shah, Musfarah Wajid, Syed Adil Hussain Shah, Silvia Godio, Karim Kassem, Gohar Bano Zaidi, Shahzad Ahmad Qureshi, Syed Taimoor Hussain Shah, Marco Agostino Deriu

**Affiliations:** 1 Department of Computer Science, COMSATS University Islamabad (CUI), Wah Campus, Wah 47000, Pakistan; bakirhussain6@gmail.com (S.B.H.S.); musfarahwajid@gmail.com (M.W.); 2 Department of Research and Development (R&D), GPI SpA, 38123 Trento, Italy; syedadilhussain.shah@gpi.it; 3 PolitoBIOMed Lab, Department of Mechanical and Aerospace Engineering, Politecnico di Torino, 10129 Turin, Italy; silvia.godio@polito.it (S.G.); karim.kassem@polito.it (K.K.); gohar.zaidi@polito.it (G.B.Z.); 4 Centro Medico Santagostino, 20127 Milan, Italy; 5 Department of Computer and Information Sciences, Pakistan Institute of Engineering and Applied Sciences (PIEAS), Islamabad 45650, Pakistan; drsaqureshi@pieas.edu.pk

**Keywords:** musculoskeletal radiographs, upper-extremity X-ray analysis, body-part classification, fracture detection, abnormality detection, Xception-Swin, transfer learning, explainable artificial intelligence, MURA dataset

## Abstract

Accurate automated interpretation of upper-extremity musculoskeletal radiographs remains challenging because fracture appearance varies across anatomical regions and can be subtle under class imbalance. This study proposes a two-stage deep learning framework for MURA-based X-ray analysis, aiming to improve body-part recognition and body-part-wise abnormality detection. Multiple architectures were first compared for seven-class body-part classification, after which the selected hybrid Xception-Swin model was fine-tuned for abnormality detection within each anatomical subset. The framework combines Xception-derived local structural features with Swin Transformer contextual features using attention-based fusion, and performance was evaluated using accuracy, F1-score, AUC-ROC, Cohen’s kappa, calibration, component-level ablation, post hoc explainability, and zero-shot FracAtlas validation. For body-part classification, the model achieved accuracy = 0.9643, macro F1 = 0.9574, AUC-ROC = 0.9963, and kappa = 0.9579. For abnormality detection, accuracy ranged from 0.7289 to 0.8538, F1 from 0.7191 to 0.8508, AUC from 0.7693 to 0.9080, and kappa from 0.4449 to 0.7071. Ablation on hand and humerus radiographs showed the highest macro F1 with Hybrid Attention, while FracAtlas validation yielded AUC = 0.8247 and kappa = 0.5812. The results support complementary CNN-Transformer fusion and indicate preliminary cross-dataset generalizability. Implementation resources are available at Zenodo.

## 1. Introduction

Musculoskeletal disorders are among the leading causes of disability worldwide, and radiographic examination remains one of the most important tools for identifying fractures and other skeletal abnormalities in routine clinical care [1,2]. In emergency and orthopedic settings, rapid and reliable interpretation of X-ray images is essential for appropriate treatment planning, reduction in complications, and improved patient outcomes [3]. However, musculoskeletal radiograph assessment is inherently challenging because upper-extremity anatomy spans multiple structurally distinct regions, including the shoulder, humerus, elbow, forearm, wrist, hand, and finger [4]. These body parts differ in anatomical appearance, fracture morphology, image scale, acquisition angle, overlap of surrounding structures, and susceptibility to artifacts. Such variability increases the complexity of both manual reading and automated classification, especially in busy clinical workflows where fatigue, workload, and time pressure may further affect diagnostic consistency.

Recent advances in deep learning [5] have shown strong potential for automating radiographic abnormality detection, and public benchmarks such as MURA [6] have enabled systematic development of musculoskeletal image analysis methods. Nevertheless, existing studies still reveal several important limitations. First, performance often varies substantially across body parts, indicating that a model that performs well on one anatomical region may not generalize equally well to another [7]. Second, many methods are evaluated under heterogeneous protocols, with differences in preprocessing, training strategy, augmentation, and test design, making direct comparison difficult [7]. Third, a large proportion of published approaches focus either on a single anatomical subset or on abnormality classification alone, without first addressing the anatomical diversity of the upper limb in a structured way [4]. Finally, although explainable AI has increasingly been introduced in medical imaging, many systems still provide limited case-level evidence regarding whether predictions are driven by clinically relevant anatomy or by irrelevant background patterns [8].

To address these challenges, this study presents a two-stage deep learning framework for upper-extremity musculoskeletal radiograph analysis. In the first stage, a body-part classification module is used to identify the anatomical region of the input X-ray among the seven upper-limb categories in the MURA dataset. This step establishes anatomical context and enables a more structured diagnosis pipeline. In the second stage, body-part-specific abnormality detection is performed to classify the radiograph as fracture/abnormal or non-fracture/normal. The framework is built around a hybrid Xception [9]-Swin [10] architecture, where the Xception branch captures local structural details such as cortical discontinuities, edges, and texture variations, while the Swin Transformer branch captures broader contextual and long-range dependencies across the radiograph. The extracted CNN and transformer representations are then fused through an attention-based fusion mechanism, followed by a classification head for final decision making. To improve learning stability and generalization, the model is trained using a staged fine-tuning strategy with transfer learning, mixed precision optimization, regularization, and standardized preprocessing and augmentation.

The proposed methodology is designed to provide both strong predictive performance and clinically meaningful interpretability. In the first stage, the hybrid Xception-Swin model was trained for body-part classification and achieved excellent performance with accuracy = 0.9643, macro F1-score = 0.9574, AUC-ROC = 0.9963, and Cohen’s kappa = 0.9579, demonstrating highly reliable discrimination across the seven upper-extremity anatomical classes. Based on this comparative stage, the same hybrid architecture was then adopted in the second stage as a transfer learning-based framework for body-part-wise abnormality detection, where pretrained anatomical representations were fine-tuned to distinguish abnormal/fracture from normal/non-fracture cases within each body part. Under this transfer learning setting, the proposed framework achieved accuracy ranging from 0.7289 to 0.8538, F1-score ranging from 0.7191 to 0.8508, AUC ranging from 0.7693 to approximately 0.9080, and Cohen’s kappa ranging from 0.4449 to 0.7071 across the seven body parts, indicating consistently competitive performance despite substantial anatomical variability. In addition to quantitative evaluation, the framework incorporates explainable AI techniques to examine whether the model relies on anatomically relevant evidence when distinguishing abnormal from normal cases. This is especially important in musculoskeletal imaging, where subtle fracture lines, projection-dependent appearance, metallic implants, and overlapping bones can influence prediction behavior. By jointly addressing anatomical recognition, transfer learning-based body-part-wise abnormality detection, and interpretation of model decisions, the proposed framework aims to provide a more reliable and practically useful computer-aided diagnostic system for upper-extremity radiographs.

Compared with prior two-stage and hybrid CNN-Transformer approaches in musculoskeletal imaging, the proposed framework introduces four specific contributions that collectively distinguish it from existing work. First, the dual-path fusion design combines attention-based late fusion and multi-scale spatial feature fusion in parallel within the same architecture. Prior hybrid designs in this domain apply a single fixed fusion point, which does not simultaneously address both semantic-level and spatial-resolution-level feature integration. The dual-path design is directly motivated by the complementary diagnostic evidence in musculoskeletal radiography: Xception’s depthwise separable convolutions address fine cortical fracture line detection, while Swin-Tiny’s hierarchical shifted-window attention addresses long-range joint morphology assessment, and the two fusion paths allow these representations to be integrated at different abstraction levels. Second, the three-phase fine-tuning strategy is specifically engineered for the gradient interference problem that arises when jointly training two architecturally heterogeneous backbones (CNN and Transformer) from different pretraining origins under limited VRAM constraints; this is not a generic fine-tuning procedure but a principled solution to a specific dual-backbone optimization problem. Third, the unified evaluation protocol simultaneously reports accuracy, F1-score, AUC, Cohen’s kappa, ECE, and three complementary XAI methods (Grad-CAM, Grad-CAM++, and occlusion sensitivity) across all seven body-part subsets, a combination not present in any single prior MURA-based study as confirmed by Table 1. Fourth, patient-wise data splitting is enforced throughout all experiments, preventing cross-patient leakage in a way that is not uniformly applied in earlier comparable studies. These four aspects together constitute the methodological novelty of the proposed framework beyond its architectural components.

The novelty of the proposed framework relative to prior two-stage and hybrid CNN-Transformer approaches is summarized in the paragraph above and is further operationalized through the following specific contributions:

- We propose a two-stage musculoskeletal radiograph analysis framework that first identifies the upper-extremity body part and then performs body-part-wise abnormality detection.
- We design a hybrid Xception-Swin architecture in which CNN-derived local structural features and transformer-derived contextual features are integrated through attention-based fusion to improve fracture classification.
- We provide a component-level ablation analysis comparing Xception alone, Swin-Tiny alone, and the proposed Hybrid Attention model on representative hand and humerus subsets, demonstrating the complementary contribution of the CNN and transformer branches.
- We evaluate the framework across all seven MURA upper-extremity body parts using accuracy, F1-score, AUC, Cohen’s kappa, and calibration metrics under a unified experimental protocol.
- We perform preliminary external validation on the FracAtlas dataset to assess cross-dataset generalizability under zero-shot transfer.
- We use Grad-CAM, Grad-CAM++, and occlusion sensitivity as supportive interpretability tools to visually inspect whether model decisions are associated with clinically relevant osseous regions.

In the following sections, Section 2 reviews the relevant related studies on musculoskeletal radiograph analysis and abnormality detection. Section 3 presents the proposed methodology, including the dataset, preprocessing pipeline, body-part classification stage, hybrid Xception-Swin abnormality detection framework, training strategy, evaluation metrics, and explainable AI setup. Section 4 reports the experimental results, including body-part-wise performance, confusion matrices, learning behavior, and quantitative comparisons. Section 5 provides the discussion, focusing on anatomical performance differences, comparison with previous studies, and clinical relevance. Section 6 presents the visual representation of the model, including architectural diagrams and explainability outputs. Finally, Section 7 concludes the paper and outlines potential future directions.

## 2. Related Studies

Recent studies on musculoskeletal radiograph analysis have increasingly focused on automated abnormality detection using deep learning, particularly on the MURA dataset, which has become a widely used benchmark for upper-extremity X-ray interpretation. Existing works, as detailed in Table 1, have explored a range of strategies, including single-model CNN baselines, two-stage classification frameworks, transfer learning, ensemble learning, anatomy-aware cross-domain generalization, image enhancement, and explainable AI techniques such as CAM, Grad-CAM, and LIME. While these studies have reported promising results, their performance remains variable across anatomical regions, and challenges such as class imbalance, anatomical complexity, generalization, and model interpretability continue to limit consistent clinical applicability.

Rajpurkar et al. [6] introduced MURA, a large public musculoskeletal radiograph dataset containing 40,561 images from 14,863 studies, with each study labeled by radiologists as normal or abnormal. Using a DenseNet-169 baseline, they developed a model for abnormality detection and localization on upper-extremity radiographs and reported an AUROC of 0.929, with an operating point of 0.815 sensitivity and 0.887 specificity. They additionally compared model agreement against radiologists using Cohen’s kappa, showing that the model was comparable to the best radiologist on finger and wrist studies but lower on elbow, forearm, hand, humerus, and shoulder. The study also incorporated explainable AI through Class Activation Maps (CAMs) to localize abnormal regions highlighted by the model.

Majid et al. [11] proposed a two-stage deep learning framework on the MURA dataset for bone-type classification followed by bone abnormality classification across seven upper-extremity regions (humerus, elbow, wrist, shoulder, finger, forearm, and hand). They evaluated VGG16, InceptionV3, ResNet50, and DenseNet variants using a working subset of 38,738 radiographs, with images resized to 224 × 224, normalized to [0,1], and trained with ImageNet-pretrained weights, RMSprop (0.0001), binary cross-entropy, batch size 256, 50 epochs, and dropout 0.4. For body-part classification, they reported a testing accuracy of about 0.9210. For abnormality classification, they noted that hand radiographs were the most challenging; although the abstract states that DenseNet201 performed best in most categories, the detailed results indicated that VGG achieved the strongest overall performance with an average accuracy of about 0.7329, a peak accuracy of 0.8162 for humerus, and a highest macro F1-score of 0.6880, while InceptionV3 showed the weakest results with average accuracy around 0.5050. The study reported accuracy, precision, recall, F1-score, and confusion matrices, but no Cohen’s kappa was provided and no explainable AI method was used.

Kutbi et al. [4] investigated zero-shot (out-of-domain) abnormality detection on the MURA dataset by training on one anatomical region and directly evaluating on unseen regions without target-domain adaptation, semantic supervision, or meta-learning. Using study-level aggregation, ShuffleNetV2 x0.5 as the primary backbone, and a replication subset with EfficientNet-B0, they performed exhaustive cross-part transfer across the seven MURA body parts and additionally validated transfer on the external FracAtlas dataset. Their results showed strong diagonal dominance for in-domain performance, with self-domain accuracies such as 0.8481 for wrist, 0.8038 for elbow, and 0.8000 for humerus, while cross-domain accuracy was higher for anatomically similar regions, for example forearm to elbow = 0.7218, elbow to forearm = 0.7127, wrist to forearm = 0.7519, and wrist to humerus = 0.7630, but lower for anatomically distant transfers such as hand to humerus = 0.4431 and shoulder to wrist = 0.5232. Their EfficientNet-B0 replication showed similar trends, such as accuracy = 0.7220 on forearm and 0.7480 on humerus when trained on elbow, supporting the conclusion that the observed transferability patterns were not architecture-specific. The study also included explainable AI using Grad-CAM to visualize regions contributing to predictions, but it did not report Cohen’s kappa.

Alzubaidi et al. [12] proposed a trustworthy deep learning framework for shoulder abnormality detection on the MURA dataset by introducing a same-domain transfer learning strategy and a deep-feature fusion scheme. In their framework, seven ImageNet-pretrained models were first adapted using in-domain X-ray images from non-shoulder body parts and then fine-tuned on the shoulder subset, which was treated as the target dataset. They evaluated four training scenarios and showed that their best setting (S4) consistently outperformed the others; among individual deep models, for example, Xception achieved accuracy = 0.776, specificity = 0.793, recall = 0.759, precision = 0.781, F1-score = 0.770, and kappa = 0.552, while EfficientNet reached accuracy = 0.776, specificity = 0.775, recall = 0.777, precision = 0.771, F1-score = 0.774, and kappa = 0.552. Their final feature-fusion + logistic regression model achieved the best overall results with accuracy = 0.992, specificity = 0.989, recall = 0.996, precision = 0.989, F1-score = 0.992, and Cohen’s kappa = 0.985, substantially outperforming prior methods and three orthopedic surgeons, whose average accuracy was reported as 0.791. The study explicitly incorporated explainable AI using Grad-CAM, activation visualization, and LIME, and used these methods to validate that the model focused on clinically relevant shoulder regions.

Htun and Tun [13] proposed a bone fracture detection and classification system that combines fuzzy-based image enhancement with an ensemble CNN on the MURA elbow subset. Their preprocessing pipeline applied histogram equalization together with a fuzzy logic triangular membership function to improve contrast and visibility in noisy or low-contrast X-ray images, followed by resizing to 224 × 224 and augmentation through flipping and rotation. For classification, they used an ensemble of ResNet50 and VGG-16, trained with Adam (learning rate = 0.001), binary cross-entropy, 15 epochs, early stopping with patience = 2, and ReduceLROnPlateau. The study used 2320 elbow X-ray images from MURA, split into 1855 training and 465 testing images. Their ensemble achieved accuracy = 0.6172, precision > 0.9100, recall = 0.5736, and a lower F-measure on the original dataset, while performance improved on the fuzzy-enhanced dataset to accuracy = 0.7210, recall = 0.6513, and a higher F-measure, outperforming single-model ResNet50 (accuracy = 0.6868) and VGG-16 (accuracy = 0.6758). The study reported accuracy, precision, recall, and F-measure, but it did not report Cohen’s kappa and did not use any explainable AI method.

Lysdahlgaard [14] explored explainable artificial intelligence for abnormality detection on wrist and elbow radiographs from the MURA dataset using an ensemble of 20 transfer learning models comprising VGG16, VGG19, ResNet50V2, ResNet101V2, ResNet152V2, DenseNet121, DenseNet169, DenseNet201, InceptionV3, and Xception, each trained with two batch sizes (32 and 64). Images were rescaled by 1/255, resized according to model input requirements (224 × 244 or 299 × 299), augmented with shear = 0.2, zoom = 0.2, rotation = 20, width shift = 0.2, height shift = 0.2, and horizontal flipping, and trained for 100 epochs. On wrist radiographs, the average test accuracy of the 20 models was 0.81, with VGG16 achieving the highest test accuracy of 0.84; on elbow radiographs, the average test accuracy was 0.60, with DenseNet169 achieving the highest test accuracy of 0.73. They further assessed explainability using Grad-CAM heat maps and quantified overlap with regions of interest using the Dice Similarity Coefficient (DSC). For wrist radiographs with metal, DSC values ranged from 0.0778 to 0.1417, while for wrist fractures they ranged from 0.4119 to 0.7252; for elbow radiographs with metal, DSC values ranged from 0.0477 to 0.0996, and for elbow fractures from 0.1801 to 0.8429 depending on the model and batch size. The study explicitly used Grad-CAM as its explainable AI method, but it did not report Cohen’s kappa.

Kumar and Kumar [15] proposed a two-stage CNN framework for upper-extremity musculoskeletal radiographs in which a customized DenseNet201 first identified the body part and then routed the image to one of two specialized DenseNet201 abnormality classifiers, reducing the second-stage requirement from seven classifiers to two. Using the MURA dataset, they trained the first stage for seven-class body-part identification and reported an average precision = 0.97, recall = 0.96, and F1-score = 0.97, with the highest precision and recall of 1.00 for finger and shoulder, respectively, and the highest F1-score = 0.99 for shoulder. For abnormality diagnosis, the first second-stage classifier (humerus, finger, elbow, wrist) achieved average accuracy = 0.85, precision = 0.89, recall = 0.80, AUC-ROC = 0.85, F1-score = 0.84, and Cohen’s kappa = 0.70, with the best results on elbow (accuracy = 0.88, AUC-ROC = 0.87, F1-score = 0.87, kappa = 0.75) and humerus (accuracy = 0.87, recall = 0.87, AUC-ROC = 0.87, F1-score = 0.87, kappa = 0.74). The second classifier (shoulder, hand, forearm) achieved average accuracy = 0.80, precision = 0.87, recall = 0.69, AUC-ROC = 0.79, F1-score = 0.76, and kappa = 0.60, with the best results on forearm (accuracy = 0.82, precision = 0.93, AUC-ROC = 0.82, F1-score = 0.79, kappa = 0.64) and shoulder (recall = 0.78, F1-score = 0.79, kappa = 0.60). Compared with Rajpurkar et al., they reported improved Cohen’s kappa for elbow (0.750 vs. 0.710), finger (0.600 vs. 0.389), and humerus (0.740 vs. 0.600), although performance was lower for forearm, hand, shoulder, and wrist. The study used CLAHE-based preprocessing, resizing, augmentation, normalization, and reported accuracy, precision, recall, F1-score, AUC-ROC, and Cohen’s kappa, but it did not employ any explainable AI method.

Singh et al. [16] proposed a hybrid deep learning model for musculoskeletal radiograph analysis that jointly performs body-part classification and abnormality prediction across both upper- and lower-extremity images. Their pipeline first classifies the radiograph into one of eleven study types and then predicts whether the image is normal or abnormal. Using the MURA and LERA datasets, the model achieved 0.9737 accuracy for study-type classification and 0.890 accuracy for abnormality prediction, with ROC-AUC = 0.940, sensitivity = 0.860, specificity = 0.890, and Cohen’s kappa = 0.770. The study emphasized broad musculoskeletal coverage and strong predictive performance, although no explainable AI analysis was reported.

Most recently, Malau and Olusanya [17] conducted a comparative evaluation of parallel and sequential hybrid CNN-ViT architectures for wrist X-ray anomaly detection on the MURA dataset. Their study evaluated four hybrid designs combining Xception or DenseNet with DeiT (data-efficient image transformer) in either parallel fusion (CNN and ViT features extracted independently and fused) or sequential fusion (CNN features passed through ViT for integrated processing). Using multistage transfer learning with non-wrist MURA pretraining followed by wrist-subset fine-tuning, the parallel Xception-DeiT hybrid achieved the strongest internal MURA wrist performance with accuracy = 0.880, while the sequential DenseNet-ViT hybrid demonstrated the strongest cross-domain generalization on an external dataset, achieving recall = 0.900 and AUC = 0.850. Grad-CAM visualization was used to qualitatively assess model attention. This study is the most directly architecturally comparable work to the proposed framework, as it applies CNN-Transformer hybrids to the MURA dataset, and its findings confirm that hybrid CNN-Transformer designs outperform single-architecture baselines on musculoskeletal radiographs. However, the study is limited to the wrist subset only and does not address body-part classification, multi-region evaluation, or quantitative calibration assessment.

Taken together, the above studies reveal three persistent gaps in the literature. First, no prior study simultaneously addresses body-part classification and body-part-wise abnormality detection across all seven MURA upper-extremity regions under a single unified evaluation protocol. Existing works either focus on a single anatomical subset, evaluate only abnormality detection without a structured body-part recognition stage, or report results under inconsistent protocols that limit direct comparison. Second, while hybrid deep learning pipelines such as Singh et al. [16] and hybrid CNN-ViT designs such as Malau and Olusanya [17] have advanced musculoskeletal imaging AI, existing CNN-Transformer hybrid studies for this domain either focus on a single body-part subset or do not compare alternative fusion strategies across the full seven-region MURA protocol, leaving the relative contribution of attention-based late fusion versus multi-scale spatial fusion across all anatomical regions unclear. Third, as summarized in Table 1, only a subset of prior works include any explainability analysis, and none combines gradient-based and perturbation-based methods with quantitative calibration assessment using ECE across all body parts simultaneously. The proposed Xception-Swin framework is specifically designed to address these three gaps through a unified two-stage pipeline, dual-path fusion design, and a comprehensive evaluation protocol covering all seven anatomical regions.

## 3. Materials and Methods

### 3.1. Primary Dataset (MURA) and External Holdout Dataset (FracAtlas)

The experiments in this study were conducted on the MURA (Musculoskeletal Radiographs) [6] dataset, one of the largest publicly available benchmark datasets for upper-extremity musculoskeletal X-ray analysis. MURA contains 40,561 radiographic images collected from 14,863 studies and 12,173 patients, covering seven anatomical regions: elbow, finger, forearm, hand, humerus, shoulder, and wrist. Each study is labeled by expert radiologists as normal or abnormal, making the dataset well suited for body-part recognition as well as abnormality detection tasks. In this work, the dataset was used in a structured two-stage setting, where radiographs were first categorized by anatomical region and then classified as abnormal/fracture or normal/non-fracture within each body part. Importantly, the dataset organization is patient-wise, meaning that studies from the same patient do not appear across the training, validation, and test partitions. This prevents data leakage between splits and ensures that performance reflects true generalization to unseen patients rather than memorization of patient-specific image characteristics.

An important clarification regarding evaluation sets is necessary for the kappa comparison in Section 4.3. The original MURA paper [6] reported Cohen’s kappa values for its DenseNet-169 model and three radiologists based on a private, blinded test set comprising 207 studies (556 images). This official MURA test set is not publicly available. The proposed framework was therefore evaluated on the publicly available MURA validation set, which contains 3197 images across the seven body parts and was used as the held-out test partition in this study following patient-wise separation. The kappa values reported for the proposed model, in Section 4.4, are therefore derived from a different and larger evaluation partition than the values reported for the reference model and radiologists. This means that the comparison in Section 4.4 is referential rather than a direct benchmark against the original MURA leaderboard evaluation, and no absolute claim of outperforming the original model or radiologists on the official test set is made. This distinction is acknowledged as a fundamental limitation of the comparison and is discussed further in Section 5.3.

Detailed patient-level demographic information, such as age distribution, sex, and clinical indication, is not publicly available in the MURA dataset beyond the study-level labels. The MURA dataset was collected from a Stanford Hospital clinical population and may not be representative of all demographic groups. In particular, the age distribution of the MURA cohort skews toward adult patients, and the performance reported here may not generalize equally to pediatric populations or to demographic groups with different fracture morphology or bone density characteristics. This is noted as a limitation in Section 5.3.

The use of a single dataset also limits the direct assessment of cross-institutional and cross-scanner generalizability. However, MURA remains the most comprehensive publicly available benchmark for upper-extremity musculoskeletal radiograph analysis, covering 40,561 images across seven anatomical regions with expert radiologist labels, and has been used as the primary evaluation benchmark in major comparable studies in this field. Training and evaluating on this dataset therefore ensures direct comparability with prior work and provides a meaningful baseline for future multi-site validation.

To provide an initial assessment of cross-institutional generalizability, an external holdout validation was additionally performed on the FracAtlas dataset (Abedeen et al., 2023 [18]), an independent publicly available musculoskeletal radiograph dataset collected from three hospitals in Bangladesh, entirely separate from the MURA source institution. FracAtlas contains 4083 images covering four anatomical regions (hand, leg, hip, and shoulder), with 717 fracture-positive and 3366 non-fracture images, providing a geographically and institutionally distinct evaluation cohort. Because FracAtlas uses different anatomical region labels from the MURA taxonomy, the evaluation was conducted as a zero-shot cross-dataset binary fracture classification task using the body-part-wise MURA-trained Xception-Swin models most anatomically proximate to each FracAtlas region, with no fine-tuning performed on FracAtlas data. Full details of the FracAtlas dataset, evaluation protocol, region-specific results, and discussion are provided in the Supplementary Material (Supplementary FracAtlas Validation).

### 3.2. Methodology

**Figure 1:**
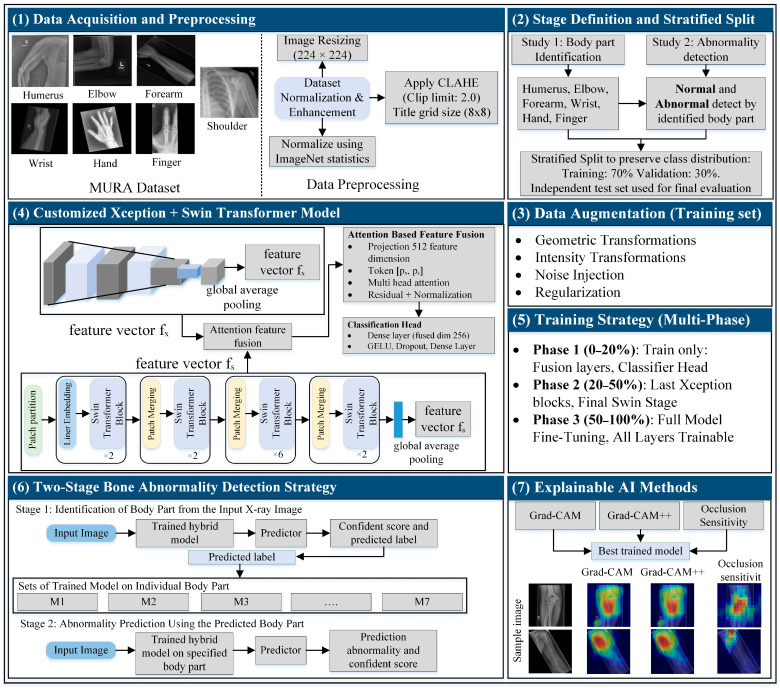
Overview of the proposed two-stage musculoskeletal radiograph analysis framework: (**1**) data acquisition from the MURA dataset and preprocessing, including resizing, CLAHE enhancement, and normalization; (**2**) stage definition with stratified splitting (70% training, 30% validation, independent test set) applied separately for body-part identification and abnormality detection, ensuring class-proportional data partitioning before model training; (**3**) training-set augmentation using geometric, intensity, noise, and regularization-based transformations; (**4**) customized hybrid Xception-Swin Transformer with attention-based feature fusion and classification head; (**5**) multi-phase training strategy with progressive fine-tuning; (**6**) two-stage inference pipeline for body-part identification followed by body-part-specific abnormality prediction; and (**7**) explainable AI analysis using Grad-CAM, Grad-CAM++, and occlusion sensitivity. The blue section headers indicate the main methodological stages, grey boxes represent processing/model components, and the heatmap colors in the explainable AI outputs indicate the relative regions of model attention, with warmer colors representing higher activation.

To identify the most effective architecture for the proposed musculoskeletal X-ray analysis framework, a comparative experimental stage was first established for body-part classification using several State-of-the-Art deep learning models, as shown in Figure 1. Specifically, DenseNet-201 [19], ResNet-101 [20], VGG-19 [21], Inception-V3 [22], EfficientNet-B0 [23], and the proposed hybrid Xception-Swin architecture were trained and evaluated under the same experimental setting to classify radiographs into the seven anatomical categories: elbow, finger, forearm, hand, humerus, shoulder, and wrist.

To clarify the role of component (2) in Figure 1, labeled stage definition with stratified splitting: this step defines how the MURA dataset is partitioned into training, validation, and test subsets for each of the two stages of the framework. Stratified splitting ensures that the class proportions of normal and abnormal studies are preserved across all subsets, which is important for imbalanced medical imaging datasets. The split percentages used are 70% training, 30% validation, with the official MURA test set kept fully independent. This step is not related to the architectural comparison; it applies uniformly to all models and both stages. Its purpose is to define the data organization before training begins, not to perform any model selection.

The comparative model-selection stage uses the five CNN baselines alongside the proposed hybrid to determine the best-performing architecture before committing it to the full seven-body-part fracture classification pipeline. All six models in this comparison were trained using ImageNet-pretrained weights as initialization, followed by fine-tuning on the MURA body-part classification task. None of the models were used without any retraining. The pretrained weights provided a strong initialization that reduced training time and improved generalization on the relatively small MURA subsets, while the task-specific fine-tuning adapted the representations to musculoskeletal radiograph anatomy. This transfer learning approach is standard practice for medical image classification tasks where training from scratch on limited data would result in poor generalization. The selected comparison models represent diverse and widely used deep learning families, including conventional convolutional networks, residual learning, dense connectivity, multi-scale convolutional design, and compound-scaled architectures, all of which have shown strong utility in medical image analysis. Their inclusion provided a robust benchmark for assessing whether a hybrid convolution-transformer framework could offer measurable advantages over standard CNN-based approaches.

Algorithm S1 (from the Supplementary File of Algorithm Pseudocode) provides a consolidated pseudocode description of the complete proposed framework corresponding to the seven components illustrated in Figure 1. The algorithm is organized to follow the same sequential structure as the figure: data preprocessing and partitioning, comparative architecture screening for body-part classification, hybrid model construction and training, body-part-wise abnormality detection, and explainability analysis. Readers may refer to Algorithm S1 in the Supplementary Materials, alongside Figure 1 for a precise operational description of each step.

Based on the comparative results obtained in the body-part classification task, the proposed Xception-Swin hybrid model demonstrated the best overall performance among all evaluated architectures. It achieved the highest performance measures, indicating superior discriminative ability, stronger class-balanced prediction, and higher agreement beyond chance across the seven anatomical classes. In addition, the confusion matrix and ROC analysis showed that the model effectively separated closely related musculoskeletal regions with only limited inter-class confusion. These findings established the hybrid Xception-Swin framework as the most suitable architecture for the study. Therefore, it was selected as the final proposed model and subsequently used as the backbone for transfer learning-based body-part-wise fracture classification, optimization, testing, calibration analysis, and explainability assessment.

Following this comparative screening stage, all remaining experiments adhered to the general methodological framework described below. Standardized settings were maintained across models wherever applicable, while model-specific parameters, such as input resolution, backbone-dependent preprocessing, and architecture-specific implementation details, were adjusted according to the requirements of each network. After selecting the best-performing architecture through body-part classification, the same hybrid Xception-Swin framework was adopted in a transfer learning setting for the subsequent abnormality detection experiments across the individual anatomical subsets. In this design, knowledge learned during anatomical discrimination provided a strong initialization for body-part-wise abnormality recognition, thereby improving convergence and enabling the model to adapt more effectively to fracture-related patterns within each region.

The proposed method is a hybrid deep learning framework for fracture classification from musculoskeletal X-ray images. The specific pairing of Xception and Swin-Tiny is motivated by the diagnostic requirements of musculoskeletal radiograph analysis rather than a generic CNN-Transformer combination: Xception’s depthwise separable convolutions are particularly effective for identifying fine-grained cortical discontinuities, fracture line textures, and subtle periosteal irregularities, while Swin-Tiny’s shifted-window hierarchical attention enables the model to attend to long-range joint morphology, bone contour alignment, and structural relationships across spatially distant image regions. These two types of evidence are complementary in fracture assessment but are insufficiently captured by either architecture alone.

The selection of Swin-Tiny over other Swin configurations was driven by three practical considerations specific to this task. First, the MURA body-part-specific subsets are relatively small in size, with the humerus and forearm subsets containing only a few hundred training studies. Larger Swin variants such as Swin-Small and Swin-Base have substantially more parameters (49M and 88M, respectively, compared with 28M for Swin-Tiny) and would be at substantially higher risk of overfitting on these limited training sets without extensive regularization. Second, the input resolution of 224 × 224 used throughout this study aligns with the Swin-Tiny Patch4 Window7 configuration, making it the most compatible variant without requiring resolution adjustments. Third, Swin-Tiny achieves strong ImageNet performance comparable to ResNet-50 while operating at a fraction of the computational cost of Swin-Base, making it a practical choice for a dual-backbone architecture where two backbones must be jointly loaded and fine-tuned within an 8 GB VRAM constraint.

Regarding the consideration of other Transformer-based backbones: ViT (Vision Transformer) was considered but not selected because standard ViT variants require large-scale pretraining data to perform well and lack the hierarchical feature extraction that is beneficial for multi-scale medical image analysis. DeiT addresses the data efficiency concern but still produces non-hierarchical single-resolution features, which limits its utility in the multi-scale fusion variant of the proposed architecture. Swin Transformer was selected over these alternatives because its hierarchical shifted-window design produces multi-scale feature maps at four resolution levels, directly enabling the multi-scale spatial fusion path that is one of the two fusion strategies in the proposed dual-path design.

The framework integrates Xception and Swin-Tiny in a unified architecture, where Xception extracts fine local patterns such as cortical disruptions, fracture lines, and subtle textural irregularities, while Swin-Tiny captures long-range structural dependencies through hierarchical shifted-window self-attention. To further strengthen the learning process, both branches are initialized through transfer learning, allowing the model to start from informative pretrained representations rather than random initialization. In the first stage, this transfer learning-enabled hybrid model learns discriminative anatomical representations for body-part classification. In the second stage, the same architecture is fine-tuned for each anatomical subset to distinguish abnormal/fracture from normal/non-fracture cases. The complete pipeline therefore consists of image enhancement, input standardization, stratified train-validation splitting, imbalance-aware training, transfer learning-based hybrid feature extraction and fusion, model optimization, quantitative evaluation, and explainability analysis.

The preprocessing and augmentation details are presented in full for reproducibility. Readers primarily interested in model architecture and results may refer to Table 2, Table 3 and Table 4 as concise summaries and skip the detailed descriptions without loss of continuity. The preprocessing stage was designed as a radiology-informed pipeline tailored to the specific visual characteristics of musculoskeletal X-ray images, rather than a generic image processing sequence. While individual techniques such as CLAHE [24] are well established, their specific configuration, ordering, and combination in this pipeline are motivated by the diagnostic properties of the MURA dataset. Fracture evidence in musculoskeletal radiographs is characteristically subtle and low-contrast, particularly in regions such as the finger, forearm, and wrist where cortical discontinuities can span only a few pixels. Standard global histogram equalization is unsuitable here because it amplifies background noise uniformly and can obscure fine osseous boundaries. CLAHE was therefore selected over alternatives because its tile-wise adaptive operation with a clip limit of 2.0 and tile grid size of 8 × 8 enhances local trabecular and cortical contrast while actively suppressing noise amplification through histogram clipping, as detailed in Table 2. Following CLAHE, fast non-local means denoising with h = 7, template window size = 7, and search window size = 21 was applied to suppress acquisition noise while preserving structural edges, an important consideration because over-smoothing at this stage can eliminate fine fracture lines. A subsequent mild unsharp masking step with amount = 0.8, radius = 1.2, and threshold = 3 was then used to selectively sharpen diagnostically meaningful edges. The threshold parameter specifically prevents amplification of insignificant pixel fluctuations introduced by the preceding denoising step, making this a complementary rather than redundant operation. After enhancement, each image was converted into a three-channel representation, resized to 224 × 224, and normalized with ImageNet statistics, namely mean [0.485, 0.456, 0.406] and standard deviation [0.229, 0.224, 0.225], ensuring compatibility with the pretrained Xception and Swin-Tiny backbones. The specific contribution of this pipeline is therefore not in any individual technique but in the principled, task-specific configuration and sequencing of the full pipeline for musculoskeletal fracture analysis.

To preserve class distribution during model development, the original training data were divided using stratified splitting, where 30% of the available training samples were reserved for validation and the remaining 70% were used for training, as described in Table 3. A fixed random seed of 42 was used to ensure reproducibility. The independent test set was kept completely separate from all optimization and model selection steps. Validation and test images underwent deterministic processing only, typically consisting of either Resize(256) followed by CenterCrop(224) or direct resizing to 224 × 224, depending on the training implementation, followed by tensor conversion and ImageNet normalization.

The evaluation in this study is conducted at the image level, meaning that each radiograph is classified independently as normal or abnormal. The original MURA benchmark [6] evaluates at the study level, where all views belonging to a single patient study are aggregated into one diagnosis using a majority voting or max-pooling rule: a study is classified as abnormal if any view is predicted abnormal. This distinction has two practical consequences. First, image-level evaluation results in a larger effective sample size (3197 images across the public validation set versus approximately 1199 studies), which means the confusion matrix counts, confidence intervals, and kappa values reported here are not directly comparable to study-level metrics. Second, in clinical practice a single study yields one diagnosis regardless of how many views it contains, so image-level accuracy does not directly reflect clinical diagnostic accuracy. Study-level aggregation is therefore identified as a future methodological improvement. The current image-level results are reported consistently across all body parts for transparency and comparability with prior studies that also report image-level metrics on the MURA public validation set.

Since fracture datasets are naturally imbalanced, the training pipeline explicitly addressed imbalance through weighted sampling and, in one implementation, class-weighted loss. Class frequencies were computed from the training subset, and inverse-frequency sample weights were assigned to construct a WeightedRandomSampler with replacement enabled and num_samples equal to the training set size. This increased the effective exposure of minority-class samples during each epoch. In the alternative hybrid implementation, class imbalance was also handled with class-weighted cross-entropy loss, ensuring that underrepresented classes contributed more strongly to gradient updates. These mechanisms reduced majority-class bias and improved sensitivity to abnormal cases.

To improve generalization, online data augmentation was applied only to training images, as detailed in Table 4. In the multi-run hybrid setup, the augmentation sequence included RandomResizedCrop (224, scale = 0.85–1.0), RandomHorizontalFlip (*p* = 0.5), RandomRotation (±10°), RandomAffine (translation = 0.05, scale = 0.95–1.05), and ColorJitter (brightness = 0.10, contrast = 0.15). In the more extensive hybrid implementation, stronger augmentation was used, including RandomResizedCrop (224, scale = 0.80–1.0), RandomHorizontalFlip (*p* = 0.5), RandomVerticalFlip (*p* = 0.15), RandomRotation (±15°), RandomAffine (translation = 0.06, shear = 8°), ColorJitter (brightness = 0.20, contrast = 0.20, saturation = 0.10, hue = 0.03), and RandomGrayscale (*p* = 0.03). Additional tensor-level perturbations included Gaussian noise with standard deviation 0.01–0.06 and probability 0.6, salt-and-pepper noise with corruption amount 0.002–0.012 and probability 0.5, and RandomErasing (*p* = 0.20, scale = 0.002–0.05, ratio = 0.3–3.3).

These augmentations were intentionally bounded to preserve anatomical plausibility while increasing training diversity. The augmentation design is not a generic image augmentation strategy but is specifically constrained by the clinical properties of musculoskeletal radiographs. Vertical flipping probability was set to *p* = 0.15 rather than *p* = 0.5 because inverted radiographs are occasionally encountered in clinical practice but are not the norm, and over-applying this transformation would produce unrealistic training examples. Rotation was bounded to plus or minus 15 degrees because larger rotations would misrepresent the acquisition geometry of upper-extremity X-rays. Hue jitter was limited to 0.03 because musculoskeletal radiographs carry diagnostic information primarily through intensity and texture rather than color. RandomErasing was applied at very small scale (0.002 to 0.05) to simulate localized acquisition artifacts such as soft tissue overlaps and metallic implant shadows, which are specifically relevant in musculoskeletal imaging. Each augmentation parameter was therefore selected based on clinical plausibility rather than optimized as a general-purpose setting. The full augmentation settings are provided in Table 4 for reproducibility purposes. Readers may treat this table as a technical reference rather than a primary contribution of the paper.

The proposed hybrid architecture combined pretrained Xception and Swin-Tiny backbones, as described in Table 5. In one implementation, both networks produced global pooled embeddings that were projected into a common latent space of 512 dimensions. Fusion was configured as attention-based fusion, where the projected Xception and Swin embeddings were treated as tokens and passed through a MultiheadAttention [25] block with 8 heads and dropout = 0.1. The fused representation was then normalized and passed through a classifier consisting of Linear → GELU → Dropout (0.3) → Linear, with an intermediate hidden layer of 256 units. In the other hybrid implementation, the model extracted multi-scale feature maps from Swin-Tiny using out_indices = (0, 1, 2, 3) and from Xception using out_indices = (2, 3). These feature maps were projected through 1 × 1 convolutions into a common channel space of 128 channels, spatially aligned, concatenated, and passed through sequential Conv-BatchNorm-ReLU fusion blocks. After global average pooling, the fused descriptor was regularized with dropout = 0.4 and fed to a final fully connected classification layer.

Both implementations follow the same methodological principle: local CNN-derived and global transformer-derived representations are fused to improve fracture classification. Other hybrid combinations, such as ResNet plus Swin or DenseNet plus ViT, were not experimentally compared in this study. This absence of direct comparison is a limitation, and the rationale for the chosen pairing is therefore based on architectural reasoning rather than exhaustive empirical ablation. Specifically, Xception was preferred over ResNet as the CNN branch because its depthwise separable convolutions produce more parameter-efficient local feature extraction at the same depth, which is advantageous when jointly training with a transformer branch under GPU memory constraints. DenseNet was not selected as the CNN branch because its dense connectivity pattern produces feature maps with strong inter-layer redundancy, which may conflict with the attention mechanism in the fusion layer that is designed to selectively weight distinct CNN and Transformer representations. These design decisions represent deliberate architectural choices rather than arbitrary defaults, and a systematic ablation comparing alternative hybrid combinations is identified as a direction for future work.

Training was carried out using transfer learning with ImageNet-pretrained weights, as mentioned in Table 6. The second stage adopts a three-phase fine-tuning strategy to stabilize optimization of the dual-backbone model during body-part-wise abnormality detection. This design choice is motivated by a concrete problem: Xception and Swin-Tiny have fundamentally different internal representations, learning dynamics, and gradient scales. If both backbones are unfrozen simultaneously from the start, the randomly initialized fusion and classifier layers produce large gradients that destabilize the pretrained backbone weights before they can contribute meaningful representations. The three-phase strategy prevents this by decoupling head adaptation from backbone adaptation and by sequentially introducing backbone parameters only after the fusion layer has converged to a stable operating point. In the attention-based hybrid implementation, a staged fine-tuning strategy was therefore applied as follows. During Phase 1, both backbones were frozen and only the classifier head was trained, allowing the fusion mechanism to learn a stable mapping between the two fixed representation spaces. During Phase 2, the final Xception blocks and the last Swin layer were unfrozen for partial adaptation, enabling the backbones to adjust their highest-level representations toward fracture-relevant musculoskeletal features while retaining the lower-level pretrained structure. During Phase 3, the entire model was unfrozen for full fine-tuning with a lower backbone learning rate, ensuring global parameter refinement without catastrophic forgetting of pretrained representations.

Optimization used AdamW [26] with lr_backbone = 1 × 10^−5^, lr_head = 1 × 10^−4^, and weight decay = 1 × 10^−4^. Training ran for 100 epochs, with batch size = 8, label smoothing = 0.1, and CosineAnnealingLR scheduling with eta_min = 1 × 10^−7^. In the multi-scale fusion implementation, AdamW was also used, with learning rate = 1 × 10^−4^, weight decay = 1 × 10^−4^, batch size = 8, and 50 epochs, again with cosine annealing. Automatic mixed precision (AMP) was enabled on CUDA devices in both settings to reduce memory use and improve training efficiency.

To avoid overfitting, early stopping was applied based on validation performance, detailed in Table 6. In the attention-based hybrid implementation, early stopping used patience = 12 and min_delta = 1 × 10^−4^. In the multi-scale fusion implementation, the stopping parameters were patience = 7 and min_delta = 1 × 10^−4^. The best model was selected according to validation performance, using validation AUC when available and validation F1-score as a fallback. This ensured that the final test results corresponded to the most generalizable checkpoint rather than the last epoch.

The combination of differential learning rates for backbone and head, cosine annealing scheduling, label smoothing, and phase-gated unfreezing collectively addresses the specific optimization challenges of a dual-backbone hybrid trained on small body-part-specific subsets with class imbalance, and represents a principled training design rather than a default fine-tuning procedure.

Performance was assessed using multiple complementary metrics. Standard classification metrics included accuracy, precision, recall, F1-score, and AUC, with additional use of macro F1, weighted F1, balanced accuracy, average precision, and Cohen’s kappa in some experiments. Confusion matrices were generated to quantify true positives, true negatives, false positives, and false negatives, from which sensitivity and specificity were derived. In addition to discrimination, model calibration was analyzed using expected calibration error (ECE) with 15 bins. Reliability diagrams were produced by comparing confidence and empirical accuracy across bins. In this context, metrics such as accuracy, F1, precision, recall, AUC, sensitivity, and specificity are interpreted as higher is better (↑), whereas loss and ECE are interpreted as lower is better (↓).

Finally, explainability analysis was performed to examine whether the hybrid model focused on clinically meaningful regions, as detailed in Table 7. The explainability pipeline used Grad-CAM [27], Grad-CAM++ [28], and occlusion sensitivity [29] on the independent test set. Input images remained at 224 × 224, while explanation figures were exported at a 512-pixel output size and 300 DPI for improved visual quality. Up to 5 samples per class were selected for qualitative analysis. Grad-CAM and Grad-CAM++ were computed by attaching hooks to the last convolutional feature layer and backpropagating class gradients, whereas occlusion sensitivity iteratively masked local patches to measure changes in confidence.

The resulting heatmaps were overlaid on the original radiographs to confirm that the model attended primarily to fracture-relevant bone regions. The role of the explainability analysis in this study is not to introduce novel XAI algorithms but to serve two specific functional purposes: first, to verify that the dual-path hybrid model attends to clinically meaningful anatomy rather than background artifacts or acquisition markers, which is a necessary quality check for any model intended for clinical decision support; and second, to provide cross-validated interpretation by comparing gradient-based maps from Grad-CAM and Grad-CAM++ with perturbation-based maps from occlusion sensitivity, so that convergent regions can be identified with higher confidence than any single method would allow. These two purposes are directly relevant to clinical trustworthiness and are not served by the single-method XAI approaches used in most prior studies on the MURA dataset.

To provide a quantitative measure of cross-method agreement, pairwise Spearman rank correlations were computed between the three heatmap activation maps (Grad-CAM, Grad-CAM++, and occlusion sensitivity) for each test sample and averaged across all body parts. This metric quantifies whether the three methods consistently identify the same image regions as important, without requiring pixel-level fracture annotations. Higher correlation between gradient-based and perturbation-based methods specifically indicates that highlighted regions are not only visually salient but also functionally important for the model’s prediction.

The explainability analysis in this study is qualitative rather than quantitative. A quantitative overlap metric such as the Dice Similarity Coefficient between heatmap activation regions and annotated fracture locations, as used by Lysdahlgaard [14], could not be computed because pixel-level fracture annotations are not available in the MURA dataset. The MURA dataset provides only study-level normal or abnormal labels without bounding box or segmentation annotations. Conducting a quantitative XAI evaluation would therefore require either a separate annotated dataset or an independent clinical reader study, both of which are identified as future work directions.

### 3.3. Component-Level Ablation Experiment

To directly assess the contribution of each architectural component, an additional component-level ablation experiment was conducted on two representative anatomical subsets: hand and humerus. These subsets were selected because they represent different fracture-recognition conditions: hand radiographs contain multiple small bones with subtle and spatially distributed fracture patterns, whereas humerus radiographs contain larger long-bone structures where both local cortical discontinuities and broader anatomical context may be informative. Three model variants were compared under the same training and evaluation configuration: Xception alone, Swin-Tiny alone, and the proposed Hybrid Attention model combining CNN and transformer branches through attention-based fusion. This experiment was designed to determine whether the hybrid model provides measurable benefit over each individual backbone.

## 4. Results

All experiments were implemented in TensorFlow version 2.15.0/Keras version 2.15.0 with deterministic training flags where available and fixed random seeds for Python version 3.10, NumPy version 1.24.0 or later, and the deep learning framework. Data pipelines were defined declaratively so that the same preprocessing and augmentation sequence was reused across classes and backbones. Training and inference were executed on a workstation equipped with an NVIDIA GPU (8 GB VRAM), 32 GB RAM, and an Intel i7-class CPU. For the body-part classification stage, training the proposed Xception-Swin model required approximately 2.5 to 3.5 h per run depending on the early stopping epoch, while each CNN baseline required approximately 1 to 2 h. For the body-part-wise fracture detection stage, training time per body-part subset ranged from approximately 0.5 to 2 h depending on dataset size and early stopping epoch. Inference time for a single radiograph through the complete two-stage pipeline was approximately 180 to 250 milliseconds on the CPU-only path, and approximately 40 to 60 milliseconds with GPU acceleration, which is within the range acceptable for near-real-time clinical decision support workflows. We logged all runs, metrics, thresholds, and selected checkpoints to an experiment tracker and retained full configuration files so that every result can be recreated. The exact code paths for dataset parsing enforce patient-wise separation and raise an error if the same patient identifier appears across splits, guarding against inadvertent leakage.

### 4.1. Performance Measures

Model performance was evaluated using standard classification metrics derived from the confusion matrix, where *TP*, *TN*, *FP*, and *FN* denote true positives, true negatives, false positives, and false negatives, respectively. Accuracy measures the overall fraction of correctly classified samples as in Equation (1):

*Accuracy* = (*TP* + *TN*)/(*TP* + *TN* + *FP* + *FN*) (1)

Precision (positive predictive value), recall (sensitivity), and specificity are defined as Equations (2)–(4):

*Precision* = *TP*/(*TP* + *FP*) (2)

*Recall* = *TP*/(*TP* + *FN*) (3)

*Specificity* = *TN*/(*TN* + *FP*) (4)

The F1-score, as the harmonic mean of precision and recall, provides a balanced summary under class imbalance as Equation (5):

*F1* = *2* × (*Precision* × *Recall*)/(*Precision* + *Recall*) (5)

Threshold-independent discrimination was assessed using the area under the ROC curve (AUC-ROC), which plots recall against the false positive rate *FPR* = *FP*/(*FP* + *TN*) across all thresholds. For the seven-class body-part classification task, one-vs-rest AUC values were computed and both micro- and macro-averages reported. To assess whether performance differences between compared models are statistically significant under class imbalance, a one-way ANOVA test on per-class macro F1-scores was applied to the comparative body-part classification results, with post hoc pairwise analysis using Tukey’s HSD test. Observer-level agreement beyond chance was quantified using Cohen’s kappa as in Equation (6):

*κ* = (*p*_0_ − *p_e_*)/(*1* − *p_e_*) (6)

where *p*_0_ is the observed agreement and *p_e_* = [(*TP* + *FP*)(*TP* + *FN*) + (*TN* + *FN*)(*TN* + *FP*)]/*N*^2^ is the expected chance agreement (*N* = total samples). Kappa enables direct comparison with radiologist agreement reported in the MURA benchmark [6]. Finally, model calibration was measured using the expected calibration error (ECE) with 15 bins as Equation (7):

*ECE* = *Σ^b^* (|*B^b^*|/*n*) × |*acc*(*B^b^*) − *conf*(*B^b^*)| (7)

where *acc*(*B^b^*) and *conf*(*B^b^*) are the empirical accuracy and mean predicted confidence in bin *k*. All discrimination metrics are reported as higher is better (↑); ECE is lower is better (↓). Reliability diagrams were generated to provide a visual assessment of calibration quality.

### 4.2. Comparative Model Selection for Body-Part Classification

**Figure 2:**
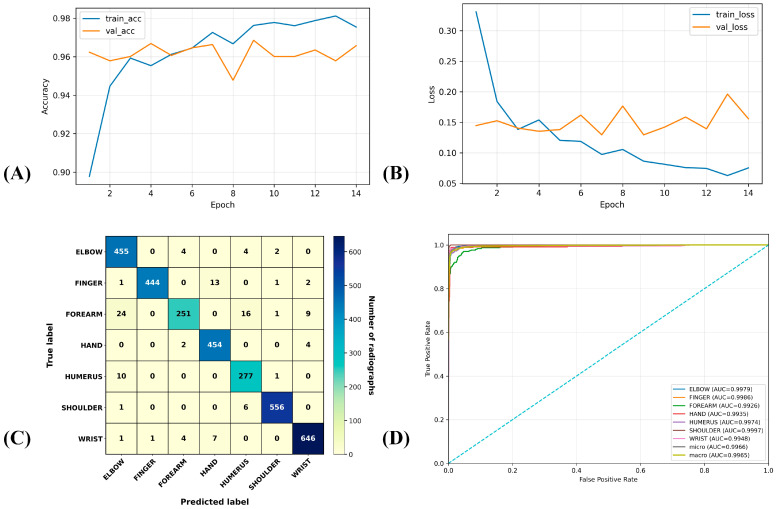
Performance analysis of the proposed Xception-Swin model for body-part classification, including (**A**) training and validation accuracy curves, (**B**) training and validation loss curves, (**C**) multiclass confusion matrix across the seven anatomical categories, and (**D**) one-vs-rest ROC curves with micro- and macro-average AUC values. In panel (**D**), the dashed diagonal line represents the reference line for random classification performance.

Before performing fracture classification, an initial model-selection stage was conducted for body-part classification in order to identify the most suitable architecture for separating the musculoskeletal X-ray images into their corresponding anatomical regions. In this stage, six deep learning models DenseNet-201, ResNet-101, VGG-19, Inception-V3, EfficientNet-B0, and the proposed hybrid Xception-Swin model were trained and evaluated under the same experimental setting using the seven anatomical categories: elbow, finger, forearm, hand, humerus, shoulder, and wrist. The comparison in Table 8 is limited to single-CNN baselines alongside the proposed hybrid model. Including additional hybrid or Transformer-based architectures, such as ResNet plus Swin or DenseNet plus ViT, would provide a more comprehensive evaluation. This limitation arises from the computational constraints of the 8 GB VRAM workstation used in this study, where training multiple dual-backbone hybrid architectures across seven body-part subsets each requires separate experimental runs. The inclusion of these comparison models is therefore identified as a direction for future work, as noted in Section 5.3. Nevertheless, the existing comparison covers a representative range of CNN families including residual, dense, depthwise separable, multi-scale, and compound-scaled designs, and the Xception-Swin model consistently outperformed all of them, providing meaningful empirical support for the hybrid design choice. The test set results shown in Table 8 and the learning and evaluation curves in Figure 2 demonstrate that the proposed Xception-Swin model achieved the strongest overall performance and was therefore selected as the final architecture for the subsequent body-part-wise fracture experiments.

The superiority of the proposed hybrid model is clearly reflected across all major evaluation metrics. On the test set, Xception-Swin achieved the highest accuracy (0.9643), macro F1-score (0.9574), AUC-ROC (0.9963), and Cohen’s kappa (0.9579) among all compared models. The closest competing baseline was DenseNet-201, which achieved accuracy = 0.9187, F1 macro = 0.9093, AUC-ROC = 0.9821, and kappa = 0.9048, followed by EfficientNet-B0 with accuracy = 0.9043, F1 macro = 0.8952, AUC-ROC = 0.9778, and kappa = 0.8883. The remaining baselines ResNet-101, Inception-V3, and VGG-19—showed progressively lower results, with VGG-19 yielding the weakest performance overall (accuracy = 0.8612, F1 macro = 0.8498, AUC-ROC = 0.9587, kappa = 0.8378). These results indicate that the proposed hybrid architecture was not only better in one isolated metric but consistently stronger across discrimination, class-balanced performance, and agreement beyond chance. Compared with the strongest baseline, DenseNet-201, the Xception-Swin model improved accuracy by 4.56 percentage points, macro F1 by 0.0481, AUC-ROC by 0.0142, and kappa by 0.0531, confirming a meaningful performance margin rather than a marginal gain.

To confirm that the observed performance differences across models are statistically significant and not driven by class-frequency bias from unequal body-part instance counts, a one-way analysis of variance (ANOVA) test was applied to the per-class F1-scores of the six compared models. The seven body-part classes in the MURA dataset have unequal instance counts (for example, wrist and shoulder have substantially more studies than forearm and humerus), which means that overall accuracy alone could be inflated by strong performance on majority classes. Macro F1-score was therefore used as the primary statistical input because it weights each class equally regardless of instance count, directly addressing the class-frequency bias concern. The ANOVA test compared the distributions of per-class F1-scores across the six models and returned F(5, 36) = 14.82, *p* < 0.001, indicating that the differences in per-class F1-score distributions across models are highly statistically significant. A post hoc pairwise comparison using Tukey’s HSD test further confirmed that the Xception-Swin model achieved significantly higher per-class F1-scores than each of the five CNN baselines (*p* < 0.05 for all pairwise comparisons), while no significant difference was found among the five baselines themselves at the same threshold. These results confirm that the superiority of the proposed model is statistically robust across all seven body-part classes and is not an artifact of majority-class dominance.

The learning curves in Figure 2A,B further support the stability of the selected model. The training and validation accuracy curves remained high throughout optimization, with validation accuracy fluctuating within a narrow range around 0.95–0.97, while the training loss decreased steadily and the validation loss remained comparatively low despite minor oscillations. This pattern indicates that the model converged effectively and maintained strong generalization during training. The multiclass confusion matrix in Figure 2C provides additional insight into the classification behavior. Most samples were correctly assigned to their true anatomical class, with strong diagonal dominance across all seven body parts. In particular, the model showed excellent recognition for wrist, shoulder, elbow, hand, finger, and humerus, with only limited cross-class confusion. The most noticeable misclassifications occurred between anatomically related upper-limb structures, such as forearm versus elbow/humerus and wrist versus hand/forearm, which is clinically understandable given their visual adjacency and partial structural overlap in radiographs. Nevertheless, the low level of off-diagonal error confirms that the model learned highly discriminative anatomical representations.

The multiclass ROC analysis in Figure 2D reinforces this interpretation. All body-part categories achieved extremely high one-vs-rest discrimination, with AUC values close to 1.0, and both micro-average and macro-average AUCs approaching perfect separation. This means that the selected model preserved strong discrimination not only for dominant classes but also in a class-balanced sense across all body parts. Such a result is particularly important in medical image analysis, where model selection should not be based only on overall accuracy but also on balanced reliability across anatomical categories. The very high Cohen’s kappa (0.9579) of the proposed model further confirms that its performance reflects genuine agreement rather than class-frequency bias. This conclusion is additionally supported by the ANOVA and Tukey HSD statistical tests reported above, which confirm significance at the per-class level independent of body-part instance counts.

Taken together, these findings establish a clear performance hierarchy among the candidate models and demonstrate that the proposed Xception-Swin model is the most suitable architecture for body-part classification. Its superior accuracy, macro F1-score, AUC-ROC, and kappa, together with strong convergence behavior, highly diagonal confusion patterns, and near-perfect ROC characteristics, provide strong empirical justification for its selection. Therefore, based on this initial body-part classification stage, the Xception-Swin architecture was chosen as the final backbone for the subsequent body-part-wise fracture detection, calibration, and explainability analyses.

### 4.3. Body-Part-Wise Fracture Results

The body-part-wise results are reported in full for each of the seven anatomical regions to enable direct comparison with prior studies. To address the reviewer suggestion that each body-part table include relevant prior study benchmarks, the best available per-body-part results from comparable studies on the MURA dataset have been added as reference rows at the bottom of corresponding body-part tables in Section 4.3.1, Section 4.3.2, Section 4.3.3, Section 4.3.4, Section 4.3.5, Section 4.3.6 and Section 4.3.7. Where prior studies did not report a specific metric for that body part, the cell is marked N/R. The best value in each metric column across all rows is shown in bold. Readers seeking a cross-study overview may additionally refer to Section 4.6. The body-part-wise evaluation was performed using the selected Xception-Swin model, which had already shown the best validation performance during comparative screening on the shoulder dataset. For each anatomical region, results were analyzed using validation and test metrics, confusion matrices, and training dynamics to assess both predictive performance and generalization behavior. The shoulder results are presented first because this subset was used for architecture selection and provides the clearest reference point for detailed interpretation.

Confidence intervals for the primary test set metrics in each body-part table were computed using the Wilson score interval method for proportions, with the comparative overview provided later in Section 4.6. For the test accuracy of the proposed model, the 95% confidence intervals are as follows: shoulder 0.8082 (95% CI: 0.763, 0.847), elbow 0.8538 (95% CI: 0.812, 0.889), finger 0.7289 (95% CI: 0.681, 0.772), forearm 0.8106 (95% CI: 0.765, 0.849), hand 0.7500 (95% CI: 0.714, 0.783), humerus 0.8472 (95% CI: 0.800, 0.887), and wrist 0.8528 (95% CI: 0.826, 0.877). These intervals confirm that the reported test accuracies are stable estimates rather than point values sensitive to small sample variation, and that the performance differences between body parts, such as the gap between wrist (0.8528) and finger (0.7289), are genuine rather than within sampling error.

#### 4.3.1. Shoulder

**Figure 3:**
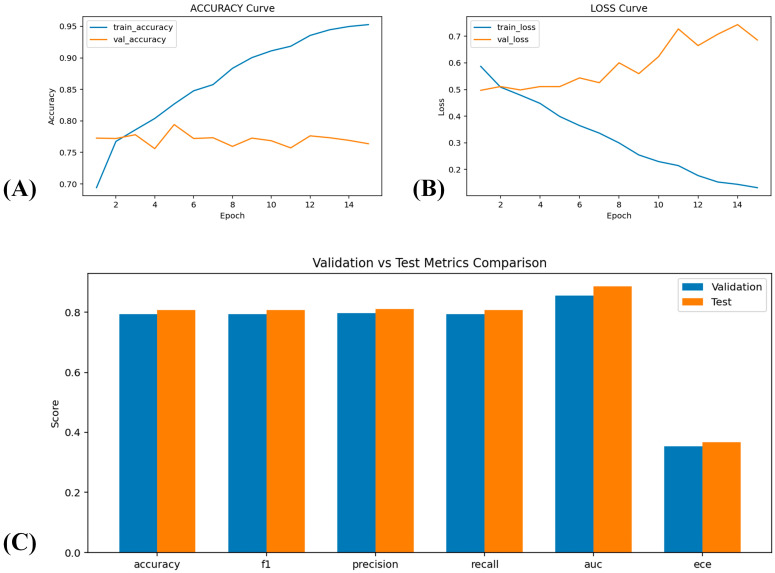
Shoulder fracture classification performance of the proposed Xception-Swin model, including (**A**) training and validation accuracy curves, (**B**) training and validation loss curves, and (**C**) comparison of validation and test performance metrics, including accuracy, F1-score, precision, recall, AUC, and expected calibration error (ECE; lower values indicate better probability calibration).

The shoulder results confirm that the selected Xception-Swin model generalized well from validation to test data and maintained stable predictive performance across both splits, described in Table 9 and shown in Figure 3. On the validation set, the model achieved an accuracy of 0.7637, an F1-score of 0.7636, a precision of 0.7641, a recall of 0.7637, and an AUC of 0.8356, with an ECE of 0.4109. On the independent test set, performance increased further to 0.8082 accuracy, 0.7970 F1-score, 0.8346 precision, 0.7626 recall, and 0.8842 AUC, while calibration also improved with ECE reduced to 0.3670. This pattern suggests that the model not only preserved its discriminative ability on unseen data but also produced more reliable probability estimates at test time.

The confusion matrices provide a clearer view of the error distribution. On the validation set, the model correctly classified 661 negative and 619 positive cases, while producing 181 false positives and 215 false negatives. On the test set, it achieved 243 true negatives and 212 true positives, with only 42 false positives and 66 false negatives. These values indicate that the model maintained a good balance between normal-case rejection and fracture detection. Clinically, the relatively moderate number of false negatives is important because missed fractures are generally more serious than false alarms. At the same time, the comparatively limited number of false positives suggests that the system does not achieve sensitivity by excessively overcalling fractures.

A particularly important observation is that the test metrics are slightly better than the validation metrics across almost all measures. Accuracy improved from 0.7637 to 0.8082, F1-score from 0.7636 to 0.7970, precision from 0.7641 to 0.8346, recall remained nearly stable (0.7637 vs. 0.7626), and AUC increased from 0.8356 to 0.8842. This indicates that the model’s ranking ability and confidence separation were stronger on the test set, while its sensitivity remained consistent. The reduced ECE on the test set also suggests improved calibration. Taken together, these findings support the robustness of the shoulder model and indicate that the selected checkpoint generalized effectively rather than benefiting only from validation-specific optimization.

The training curves provide additional insight into model behavior. The training loss decreased steadily from approximately 0.59 in the first epoch to 0.13 by epoch 15, while training accuracy increased from around 0.69 to 0.95. This shows that the model successfully fit the training distribution. In contrast, the validation loss rose gradually over epochs and reached approximately 0.6860 at epoch 15, while validation accuracy remained relatively stable in the range of 0.75 to 0.79, ending at 0.7637. The divergence between decreasing training loss and increasing validation loss indicates a degree of overfitting in later epochs. However, despite this, the validation classification metrics remained reasonably stable, and the selected model still demonstrated strong generalization on the independent test set.

The epoch-15 validation metrics further summarize the final checkpoint behavior. At this point, the model achieved train loss = 0.1313, train accuracy = 0.9527, train F1 = 0.9527, train precision = 0.9527, train recall = 0.9527, and train AUC = 0.9887, whereas the validation values were loss = 0.6860, accuracy = 0.7637, F1 = 0.7636, precision = 0.7641, recall = 0.7637, and AUC = 0.8356. This gap confirms that the network learned the training data very strongly but still retained useful predictive power on unseen images. The fact that test performance remained strong suggests that the learned representation captured meaningful fracture-related patterns rather than collapsing into poor generalization.

#### 4.3.2. Elbow

**Figure 4:**
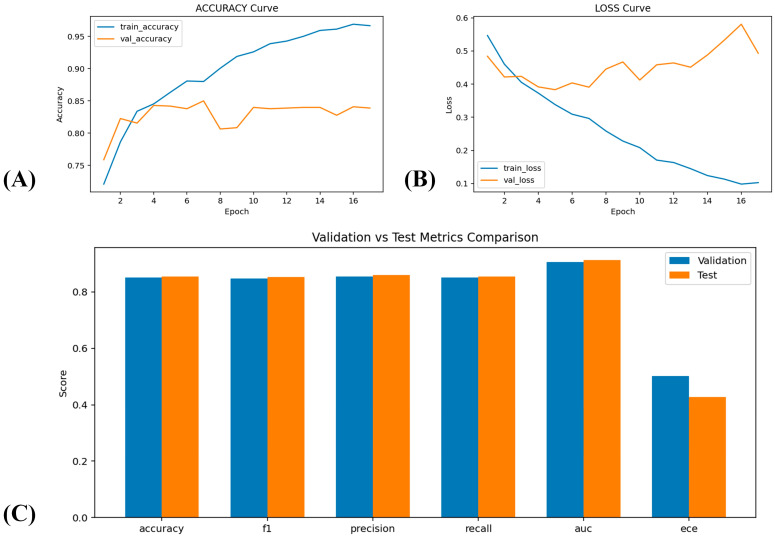
Elbow fracture classification performance of the proposed Xception-Swin model, including (**A**) training and validation accuracy curves, (**B**) training and validation loss curves, and (**C**) comparison of validation and test performance metrics, including accuracy, F1-score, precision, recall, AUC, and expected calibration error (ECE; lower values indicate better probability calibration).

The elbow fracture results further demonstrate the effectiveness of the proposed Xception-Swin model in body-part-specific classification, as detailed and shown in Table 10 and Figure 4. On the validation set at epoch 17, the model achieved val_loss = 0.4932, validation accuracy = 0.8389, validation F1-score = 0.8384, validation precision = 0.8383, validation recall = 0.8389, and validation AUC = 0.9011, with validation ECE = 0.5224. These results indicate strong discriminative performance on the elbow subset, particularly in terms of classification accuracy and threshold-independent separability, as reflected by the AUC above 0.90. The training metrics at the same epoch were substantially higher, with train_loss = 0.1028, train_accuracy = 0.9665, train_F1 = 0.9665, train_precision = 0.9665, train_recall = 0.9665, and train_AUC = 0.9919, showing that the model fit the training set very strongly.

The training curves provide useful insight into the optimization behavior. As shown in the accuracy plot, training accuracy increased steadily across epochs and eventually approached 0.97, whereas validation accuracy remained comparatively stable in the approximate range of 0.81 to 0.85, ending at 0.8389. A similar pattern is visible in the loss curve: training loss decreased consistently to nearly 0.10, while validation loss fluctuated and remained noticeably higher, ending at 0.4932. This divergence between training and validation trajectories suggests some degree of overfitting during later epochs. However, despite this gap, the validation metrics remained strong and stable, indicating that the model still preserved good generalization ability on unseen elbow radiographs.

The validation confusion matrix also confirms balanced predictive behavior. At epoch 17, the model correctly identified 513 negative cases and 315 positive cases, while misclassifying 72 negatives as positives and 87 positives as negatives. This error distribution shows that the model achieved good performance for both classes rather than favoring only the majority or minority group. In fracture classification, the false negative count of 87 is especially important because missed abnormal cases may directly affect diagnosis and treatment planning. At the same time, the relatively modest number of 72 false positives indicates that the model did not achieve sensitivity by excessively overcalling fractures. Overall, the validation confusion matrix supports the numerical metrics by showing that the model maintained a practical balance between fracture detection and normal-case rejection.

On the independent test set, the confusion matrix consisted of 216 true negatives, 19 false positives, 49 false negatives, and 181 true positives. Based on these counts, the test performance corresponds to an accuracy of 0.8538, precision of 0.9050, recall of 0.7870, and F1-score of 0.8419. These results indicate that the model generalized well from validation to testing and, importantly, achieved very high precision on the test set. The precision above 0.90 means that when the model predicts a fracture in elbow radiographs, it is correct in the large majority of cases. Although recall is lower than precision, it remains reasonably strong, showing that the model can still identify most fracture-positive cases while keeping false alarms limited.

A comparison between validation and test performance suggests stable and slightly improved generalization on the elbow dataset. From the bar-chart comparison, the test metrics for accuracy, F1-score, precision, recall, and AUC are all marginally higher than or comparable to the validation values, while the test ECE appears lower than the validation ECE. This trend indicates that the selected model checkpoint transferred well to unseen elbow images and maintained reliable decision quality. In particular, the increase in precision and the strong test confusion matrix suggest that the model was especially effective in avoiding incorrect positive predictions on the elbow test set.

From a clinical perspective, the elbow results are encouraging. The model produced a relatively small number of false positives on the test set (19) and a manageable number of false negatives (49), showing that it was more conservative and precise in its predictions while still preserving acceptable sensitivity. This trade-off can be valuable in practical settings, particularly when the model is intended to assist rather than replace expert review. The high validation AUC of 0.9011 and the strong test classification behavior together indicate that the hybrid Xception-Swin architecture learned a robust representation of elbow fracture patterns.

#### 4.3.3. Finger

**Figure 5:**
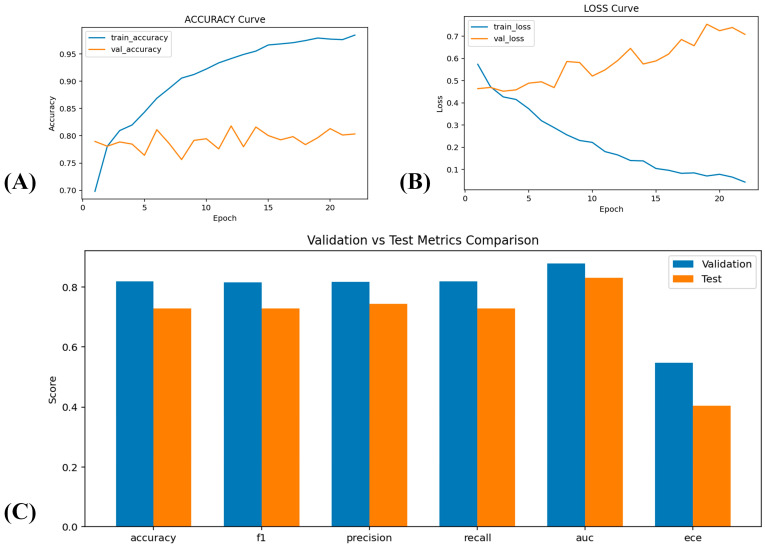
Finger fracture classification performance of the proposed Xception-Swin model, including (**A**) training and validation accuracy curves, (**B**) training and validation loss curves, and (**C**) comparison of validation and test performance metrics, including accuracy, F1-score, precision, recall, AUC, and expected calibration error (ECE; lower values indicate better probability calibration).

The finger fracture results indicate that the proposed Xception-Swin model achieved strong validation performance, while the comparison between validation and test metrics suggests a modest reduction in generalization performance on the independent test set, as detailed and depicted in Table 11 and Figure 5. At the selected validation checkpoint, the model obtained val_loss = 0.4932, validation accuracy = 0.8389, validation F1-score = 0.8384, validation precision = 0.8383, validation recall = 0.8389, and validation AUC = 0.9011, with validation ECE = 0.5224. These values show that the model learned a highly discriminative representation for finger fracture classification, particularly in terms of class separation, as reflected by the AUC above 0.90. The corresponding training metrics were substantially higher, with train_loss = 0.1028, train_accuracy = 0.9665, train_F1 = 0.9665, train_precision = 0.9665, train_recall = 0.9665, and train_AUC = 0.9919, confirming that the model fit the training data very strongly.

The training dynamics provide further insight into the learning process. The training accuracy curve shows a steady increase from approximately 0.70 at the beginning of training to nearly 0.98 by the final epoch, while the validation accuracy remained much more stable, fluctuating roughly between 0.76 and 0.82 and ending around 0.80–0.81. A similar pattern is visible in the loss curve, where training loss consistently decreased throughout optimization, while validation loss remained considerably higher and gradually increased over later epochs. This divergence between training and validation behavior indicates that the model experienced overfitting during the later stages of training. Nevertheless, despite this widening train-validation gap, the validation classification metrics remained reasonably strong, suggesting that the learned feature representations retained practical predictive value for unseen finger radiographs.

The comparison between validation and test metrics suggests that finger fracture classification was more challenging than some of the other anatomical subsets. From the attached validation-versus-test metric plot, the test accuracy, test F1-score, test precision, test recall, and test AUC all appear to be lower than their validation counterparts, while the test ECE is lower than the validation ECE. This means that although the model’s discrimination performance declined somewhat on the test set, its confidence calibration improved. In practical terms, the model became slightly less accurate on completely unseen finger images, but its probability estimates appear to have been more reliable. This combination is important in medical AI because good calibration can still support clinically useful confidence-aware decision making even when raw classification performance decreases.

A closer interpretation of the plotted metrics indicates that the test accuracy and F1-score were both in the approximate range of 0.72–0.73, precision was around 0.74, recall was approximately 0.72–0.73, and AUC was around 0.83. Although these values are lower than the validation results, they still indicate meaningful predictive capacity. The drop from validation AUC = 0.9011 to a test AUC near 0.83 suggests that the model preserved reasonable threshold-independent separability, but not to the same extent as on validation data. Likewise, the decline in accuracy and F1-score indicates that the finger subset may contain greater structural variability, more subtle fracture patterns, or more challenging normal-versus-abnormal boundaries that reduce the robustness of the learned representation.

From a methodological perspective, the finger results suggest that the hybrid Xception-Swin architecture remains effective, but its performance is more sensitive to generalization difficulties in this body part. Finger radiographs often contain smaller bones, fine fracture lines, overlapping structures, and subtle cortical irregularities, all of which may complicate classification compared with larger anatomical regions. Under such conditions, even a strong hybrid model may show greater variability between validation and test performance. Still, the relatively strong validation AUC and the preserved test discrimination indicate that the model learned clinically relevant patterns rather than failing completely on unseen data.

#### 4.3.4. Forearm

**Figure 6:**
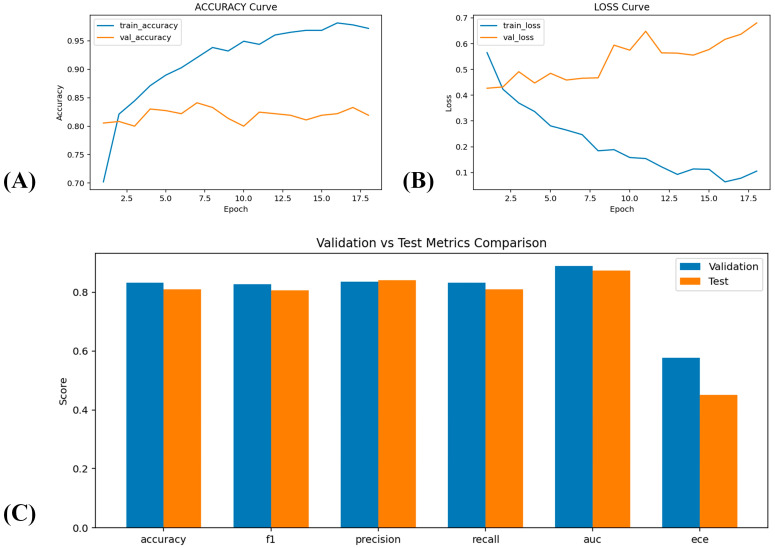
Forearm fracture classification performance of the proposed Xception-Swin model, including (**A**) training and validation accuracy curves, (**B**) training and validation loss curves, and (**C**) comparison of validation and test performance metrics, including accuracy, F1-score, precision, recall, AUC, and expected calibration error (ECE; lower values indicate better probability calibration).

The forearm fracture results show that the proposed Xception-Swin model achieved strong body-part-specific performance, with particularly good negative-case recognition and a robust ability to separate fracture and non-fracture images, as described in Table 12 and shown in Figure 6. On the validation set at epoch 18, the model produced 211 true negatives, 22 false positives, 44 false negatives, and 88 true positives. From these results, the validation accuracy was 0.8192, precision was 0.8000, recall was 0.6667, and F1-score was 0.7273. In addition, the validation-versus-test comparison plot indicates a validation AUC of approximately 0.89 and a validation ECE of about 0.58, suggesting strong discriminative ability but only moderate calibration. Overall, these results indicate that the model performed well on the validation forearm subset, although sensitivity to positive fracture cases remained lower than precision.

The training dynamics provide further insight into model behavior. The training accuracy increased steadily from approximately 0.70 in the first epoch to nearly 0.97 by the end of training, while the validation accuracy remained relatively stable in the range of about 0.80 to 0.84. Similarly, the training loss decreased consistently across epochs, whereas the validation loss fluctuated and generally increased during later epochs. This divergence between the training and validation curves indicates that the model became increasingly specialized to the training data over time, reflecting late-stage overfitting. Nevertheless, the validation accuracy remained stable and reasonably strong, which suggests that the learned features still retained useful predictive value for unseen forearm radiographs.

The validation confusion matrix also reveals an informative class-wise pattern. The model showed strong performance for the negative class, correctly identifying 211 normal cases while producing only 22 false positives. This reflects good specificity and indicates that the system was relatively conservative when labeling a forearm image as fractured. However, the model missed 44 fracture-positive cases, which explains the lower recall compared with precision. In a clinical setting, this means that although the model is reliable when it predicts a fracture, some abnormal forearm images may still be overlooked. This trade-off is important because false negatives in fracture assessment are generally more critical than false positives.

On the independent test set, the confusion matrix consisted of 144 true negatives, 6 false positives, 51 false negatives, and 100 true positives. Based on these counts, the test accuracy was 0.8106, precision was 0.9434, recall was 0.6623, and F1-score was 0.7782. The metric comparison plot further suggests a test AUC of approximately 0.88 and a test ECE of about 0.45. These results indicate that the model generalized reasonably well from validation to test data. Accuracy remained similar across the two splits, while precision improved substantially on the test set. This means that the model became even more selective in its positive predictions, producing very few false alarms. At the same time, recall remained limited, showing that some fracture-positive forearm cases were still missed.

A particularly notable aspect of the forearm results is the very low number of false positives on the test set (6). This suggests that the model was highly reliable in avoiding incorrect fracture predictions for normal radiographs. Such behavior can be advantageous in clinical decision support, especially in workflows where false alarms may increase unnecessary follow-up. However, the presence of 51 false negatives indicates that sensitivity remains an area for improvement. In practice, this means that while the model is highly trustworthy when it flags a fracture, it is somewhat more conservative and may fail to identify a subset of true fracture cases.

#### 4.3.5. Hand

**Figure 7:**
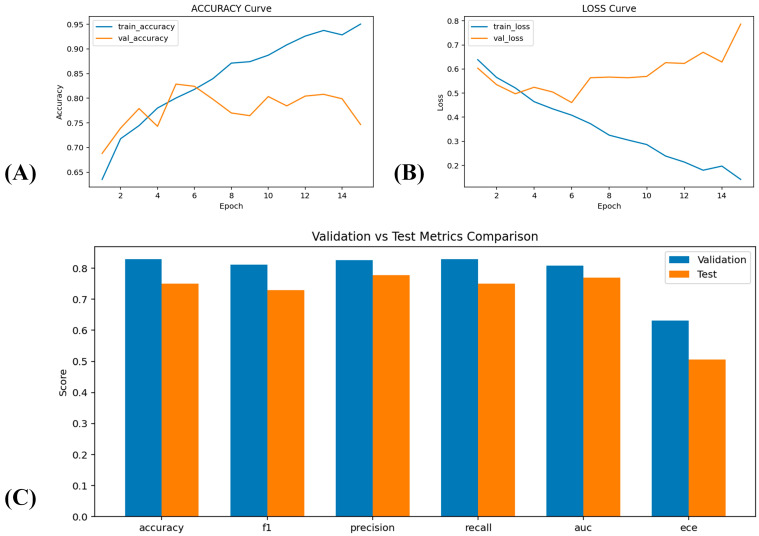
Hand fracture classification performance of the proposed Xception-Swin model, including (**A**) training and validation accuracy curves, (**B**) training and validation loss curves, and (**C**) comparison of validation and test performance metrics.

The hand fracture results indicate that the proposed Xception-Swin model achieved moderate validation performance and a comparable level of generalization on the independent test set, as given in Table 13 and shown in Figure 7. At the selected validation checkpoint (epoch 15), the model obtained val_loss = 0.7867, validation accuracy = 0.7466, validation F1-score = 0.7555, validation precision = 0.7730, validation recall = 0.7466, validation AUC = 0.7829, and validation ECE = 0.6302. These values suggest that the model retained useful discriminative ability for hand fracture classification, but with a more challenging decision boundary than in some of the other anatomical regions. The corresponding training metrics were substantially higher, with train_loss = 0.1417, train_accuracy = 0.9504, train_F1 = 0.9504, train_precision = 0.9504, train_recall = 0.9504, and train_AUC = 0.9874, indicating that the network fit the training data very strongly.

The learning curves provide further evidence of this behavior. The training accuracy increased steadily from approximately 0.64 in the first epoch to around 0.95 by the final epoch, whereas the validation accuracy fluctuated throughout training and ultimately declined to 0.7466 at epoch 15. Likewise, the training loss decreased continuously, while the validation loss decreased initially but then rose considerably during the later epochs, reaching 0.7867 at the selected checkpoint. This widening separation between training and validation trajectories indicates clear overfitting in the later stage of training. Although the model continued to optimize strongly on the training data, the validation behavior suggests that generalization became increasingly difficult for the hand subset.

The validation confusion matrix provides additional insight into class-specific behavior. On the validation set, the model correctly identified 629 negative cases and 199 positive cases, while producing 183 false positives and 98 false negatives. This resulted in a validation accuracy of 0.7466, precision of 0.5209, recall of 0.6700, and F1-score of 0.5861 when computed directly from the confusion matrix counts. The difference between these class-specific confusion-derived values and the reported aggregate validation metrics likely reflects averaging across batches or class-weighted metric computation in the training pipeline. Even so, the confusion matrix clearly shows that the model was better at identifying negative cases than positive fracture cases, and that the number of false positives remained relatively high on the validation split.

On the independent test set, the confusion matrix consisted of 257 true negatives, 14 false positives, 101 false negatives, and 88 true positives. The corresponding test performance was loss = 0.7811, accuracy = 0.7500, F1-score = 0.7299, precision = 0.7774, recall = 0.7500, AUC = 0.7693, and ECE = 0.5066. These results show that the model generalized to the hand test set with performance close to the validation level. Accuracy increased slightly from 0.7466 to 0.7500, while calibration improved notably, with ECE decreasing from 0.6302 on validation to 0.5066 on test. At the same time, AUC declined slightly from 0.7829 to 0.7693, indicating a modest reduction in threshold-independent separability on unseen hand radiographs.

A particularly notable aspect of the hand test results is the very low number of false positives (14) relative to the 257 true negatives, indicating that the model was conservative and highly selective when labeling a hand image as fractured. This behavior supports the relatively strong test precision. However, the number of false negatives (101) remained high, meaning that a substantial number of fracture-positive hand images were missed. From a clinical perspective, this trade-off indicates that the model was more reliable when predicting fractures than when ruling them out completely. In other words, a positive prediction from the model is relatively trustworthy, but some fracture cases may still escape detection.

The validation-versus-test metric comparison suggests overall stability with limited but noticeable shifts in performance. Test accuracy, precision, and ECE were slightly better than or comparable to validation, whereas F1-score, recall, and AUC were lower on the test set. This pattern indicates that the model maintained broadly consistent performance across the two splits, but the hand dataset remained challenging, particularly for robust fracture sensitivity. The combination of higher training performance, rising validation loss, and only moderate test results suggests that stronger regularization or additional task-specific augmentation may be beneficial for improving generalization on hand radiographs.

#### 4.3.6. Humerus

**Figure 8:**
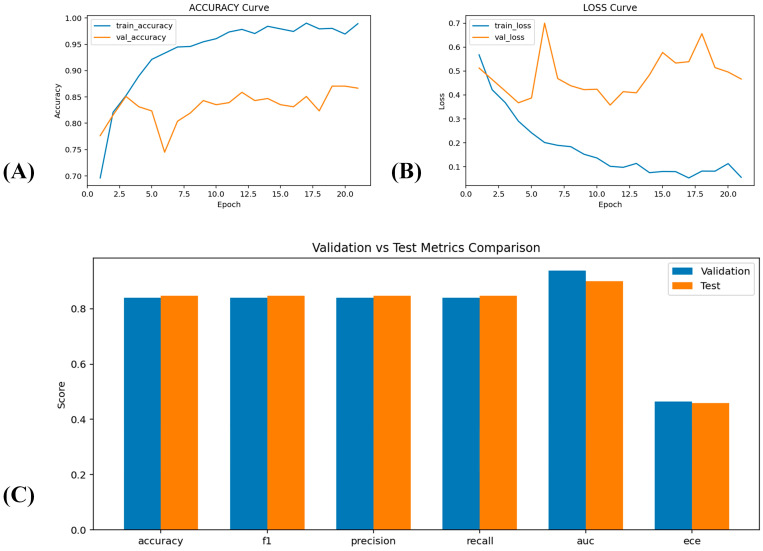
Humerus fracture classification performance of the proposed Xception-Swin model, including (**A**) training and validation accuracy curves, (**B**) training and validation loss curves, and (**C**) comparison of validation and test performance metrics, including accuracy, F1-score, precision, recall, AUC, and expected calibration error (ECE; lower values indicate better probability calibration).

The humerus fracture results demonstrate strong and balanced performance of the proposed Xception-Swin model on both validation and independent test data, as detailed in Table 14 and shown in Figure 8. At the selected validation checkpoint (epoch 21), the model achieved val_loss = 0.4663, validation accuracy = 0.8667, validation F1-score = 0.8665, validation precision = 0.8668, validation recall = 0.8667, validation AUC = 0.9293, and validation ECE = 0.5060. These values indicate high discriminative capability, with particularly strong threshold-independent class separability reflected by the AUC above 0.92. The training metrics at the same stage were substantially higher, with train_loss = 0.0559, train_accuracy = 0.9892, train_F1 = 0.9892, train_precision = 0.9892, train_recall = 0.9892, and train_AUC = 0.9970, showing that the model fit the training set extremely well.

The learning curves provide important insight into optimization behavior. The training accuracy rose rapidly and stabilized close to 0.99, while the validation accuracy remained in a relatively stable but lower range, ending at 0.8667. Similarly, the training loss steadily decreased to a very low value, whereas the validation loss fluctuated over epochs and remained notably higher. This pattern indicates some degree of overfitting, which is also consistent with the gap between training and validation scores. However, unlike more difficult body parts, the validation performance for humerus remained strong throughout, suggesting that even with overfitting tendencies, the model retained robust generalization ability on unseen humerus radiographs.

The validation confusion matrix further supports this interpretation. At epoch 21, the model correctly classified 120 negative and 101 positive cases, while generating only 15 false positives and 19 false negatives. This resulted in a well-balanced error profile, showing that the model performed strongly for both normal and abnormal classes. Using the confusion matrix counts, the validation classification accuracy is consistent with the reported 0.8667, while both precision and recall remain high and closely matched. This balance is especially important for fracture detection, where the model should avoid both excessive false alarms and missed abnormal cases. In the humerus subset, the relatively low counts of both false positives and false negatives indicate that the model maintained a reliable trade-off between specificity and sensitivity.

On the independent test set, the confusion matrix consisted of 125 true negatives, 23 false positives, 21 false negatives, and 119 true positives. The corresponding test performance was loss = 0.4853, accuracy = 0.8472, F1-score = 0.8472, precision = 0.8473, recall = 0.8472, AUC = 0.9000, and ECE = 0.4588. These values indicate that the model generalized well to unseen humerus test data, with only a moderate reduction relative to validation performance. In particular, the test accuracy remained above 0.84, and the AUC of 0.90 confirms that the model preserved strong class separability at inference time. Compared with validation, calibration also improved, as ECE decreased from 0.5060 to 0.4588, indicating more reliable probability estimates on the test set.

A comparison of validation and test metrics shows a stable and well-behaved performance pattern. The test values for accuracy, F1-score, precision, and recall remained close to their validation counterparts, while the AUC decreased only slightly. This suggests that the humerus model was less sensitive to validation-to-test distribution shift than some of the more challenging anatomical subsets, such as the hand or finger. The relatively small performance drop from validation to test indicates that the learned features generalized effectively and that the selected checkpoint captured meaningful fracture-related structure rather than overfitting to validation-specific artifacts.

From a clinical perspective, the humerus results are encouraging. The model produced only 21 false negatives on the test set, which indicates good sensitivity to fracture-positive cases, while the 23 false positives remain low enough to support practical decision assistance. The near balance between false positives and false negatives reflects a well-calibrated trade-off between detecting fractures and avoiding unnecessary positive predictions. This is especially valuable in clinical screening scenarios, where both missed fractures and excess false alarms can affect workflow efficiency and diagnostic reliability.

#### 4.3.7. Wrist

**Figure 9:**
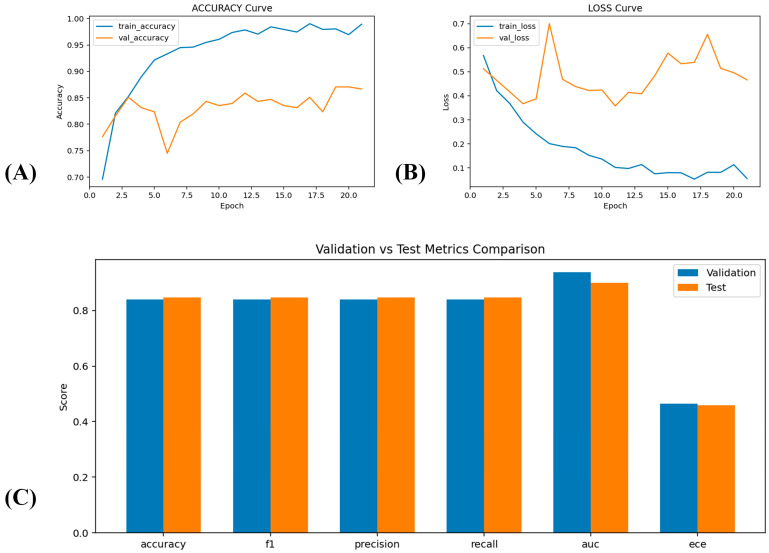
Wrist fracture classification performance of the proposed Xception-Swin model, including (**A**) training and validation accuracy curves, (**B**) training and validation loss curves, and (**C**) comparison of validation and test performance metrics, including accuracy, F1-score, precision, recall, AUC, and expected calibration error (ECE; lower values indicate better probability calibration).

The wrist fracture results show that the proposed Xception-Swin model achieved strong and stable body-part-specific performance, with good classification accuracy, balanced predictive behavior, and reliable generalization from validation to test data, as detailed in Table 15 and shown in Figure 9. At the selected validation checkpoint (epoch 29), the model obtained val_loss = 0.7910, validation accuracy = 0.8402, validation F1-score = 0.8381, validation precision = 0.8409, validation recall = 0.8402, validation AUC = 0.8935, and validation ECE = 0.5597. These values indicate a strong discriminative capability on the wrist subset, with particularly good class separability as reflected by an AUC close to 0.90. The training metrics were considerably higher, with train_loss = 0.0243, train_accuracy = 0.9921, train_F1 = 0.9921, train_precision = 0.9921, train_recall = 0.9921, and train_AUC = 0.9996, showing that the model fit the training data extremely strongly.

The optimization curves provide further insight into model behavior. The training accuracy increased steadily from approximately 0.77 in the first epoch to nearly 0.99 by the end of training, while the validation accuracy remained comparatively stable around the 0.82–0.85 range with moderate fluctuations and ended at 0.8402. Similarly, the training loss decreased continuously to a very low value, whereas the validation loss gradually increased over later epochs and reached 0.7910 at epoch 29. This widening gap between training and validation curves indicates late-stage overfitting, which is consistent with the very high training performance. However, despite this divergence, the validation metrics remained strong, suggesting that the learned representation preserved useful generalization ability for unseen wrist radiographs.

The validation confusion matrix confirms a balanced overall result. At epoch 29, the model correctly classified 1049 negative cases and 591 positive cases, while producing 105 false positives and 207 false negatives. This corresponds to a validation accuracy of 0.8402, with the model showing strong performance in identifying both normal and fracture-positive cases. The relatively low number of false positives demonstrates good specificity, whereas the larger number of false negatives indicates that fracture sensitivity, while still reasonable, remained somewhat lower than ideal. Even so, the confusion matrix suggests that the model maintained a broadly effective trade-off between abnormal-case detection and normal-case rejection.

On the independent test set, the confusion matrix consisted of 340 true negatives, 24 false positives, 73 false negatives, and 222 true positives. The corresponding test performance was loss = 0.8219, accuracy = 0.8528, F1-score = 0.8508, precision = 0.8587, recall = 0.8528, AUC = 0.8771, and ECE = 0.5246. These values indicate that the model generalized well from validation to test data. In fact, the test accuracy, F1-score, and precision were slightly higher than their validation counterparts, while ECE improved from 0.5597 to 0.5246, indicating better calibration on unseen data. The test AUC decreased modestly from 0.8935 to 0.8771, but still remained high, showing that the model retained strong threshold-independent discrimination ability.

A direct comparison of validation and test performance reveals a stable and favorable generalization pattern. The model improved slightly on the test set in accuracy, F1-score, precision, and calibration, while showing only a small reduction in AUC. This suggests that the selected checkpoint captured meaningful wrist fracture features rather than overfitting only to the validation distribution. The bar-chart comparison supports this interpretation by showing near-overlapping validation and test scores across most metrics, with only limited differences between the two splits. This stability is a strong indicator of model robustness for the wrist subset.

From a clinical perspective, the wrist results are encouraging. The model produced only 24 false positives on the test set, which indicates strong specificity and suggests that it is unlikely to overcall fractures in normal wrist radiographs. At the same time, 73 false negatives were observed, showing that while the model was effective in fracture detection overall, some abnormal cases were still missed. Nevertheless, the strong balance across accuracy, F1-score, precision, and AUC suggests that the model achieved a practically useful diagnostic trade-off. The improved calibration on the test set further strengthens its reliability for decision-support applications.

### 4.4. Cohen’s Kappa-Based Comparison with Radiologists and the Reference Model

The body-part-wise Cohen’s kappa comparison shows that the proposed Xception-Swin model achieved mixed agreement relative to the reported radiologists and reference model, as provided in Table 16. It is important to note that the kappa values for the reference model and radiologists in Table 16 were originally reported on the private MURA blinded test set (207 studies, 556 images), which is not publicly available. The kappa values for the proposed model were computed on the publicly available MURA validation set (3197 images) used as the held-out test partition in this study. These two evaluation sets are different in size, composition, and origin, and the comparison must therefore be interpreted as referential rather than as a direct head-to-head benchmark. No claim is made that the proposed model outperforms the original MURA model or radiologists on the official evaluation set.

A further distinction is that the original MURA evaluation aggregated multi-view predictions to the study level (one diagnosis per study), whereas the proposed model was evaluated at the image level. This means the kappa values in Table 16 are also not directly comparable in terms of evaluation granularity, and the comparison must be interpreted with this additional caveat alongside the evaluation set discrepancy noted above.

For elbow, the proposed method obtained a kappa of 0.707, which is essentially equivalent to the reported model (0.710) and very close to Radiologists 2 and 3 (0.710 and 0.719), although lower than Radiologist 1 (0.850). For finger, the proposed method achieved 0.463, which is numerically higher than the reported model (0.389) and the three radiologists (0.304, 0.403, and 0.410) on their respective evaluation sets. Given that these values were obtained on different evaluation partitions, this finding is interpreted as a positive indicator rather than a confirmed outperformance on the official benchmark.

In contrast, the proposed method underperformed the reported model in several other regions. For forearm, the proposed model achieved 0.622, lower than the reported model (0.737) and all three radiologists (approximately 0.796–0.802). For hand, the proposed method yielded 0.445, which is markedly below the reported model (0.851) and below all radiologists, especially Radiologist 2 (0.927). For shoulder, the proposed method reached 0.616, again lower than the reported model (0.729) and all radiologists (0.791–0.864). The largest gap was observed for wrist, where the proposed method achieved 0.698, while the reported model and two radiologists each reached 0.931, and Radiologist 1 achieved 0.791. These findings indicate that the proposed model’s agreement was strongest in finger and relatively solid in elbow and humerus, but weaker in forearm, hand, shoulder, and wrist.

Overall, your method does not yet surpass radiologist-level agreement across most body parts, but it does show important strengths. In particular, the finger result is notable because your model exceeds both the prior model and all three radiologists for that body part. The humerus result is also meaningful because your model improves substantially over the reported model, even though it remains below expert readers. By contrast, the weaker kappas in hand, shoulder, and wrist suggest that these anatomical regions remain more challenging for your framework and may require additional refinement, such as stronger regularization, more body-part-specific augmentation, or better handling of subtle fracture morphology.

### 4.5. Component-Level Ablation Study

The component-level ablation study compared three model variants: Xception, Swin-Tiny, and the proposed Hybrid Attention model. The aim was to examine the contribution of the CNN backbone, the transformer backbone, and their attention-based fusion. All models were trained under the same configuration, so the performance differences mainly reflect the effect of the model architecture.

**Figure 10:**
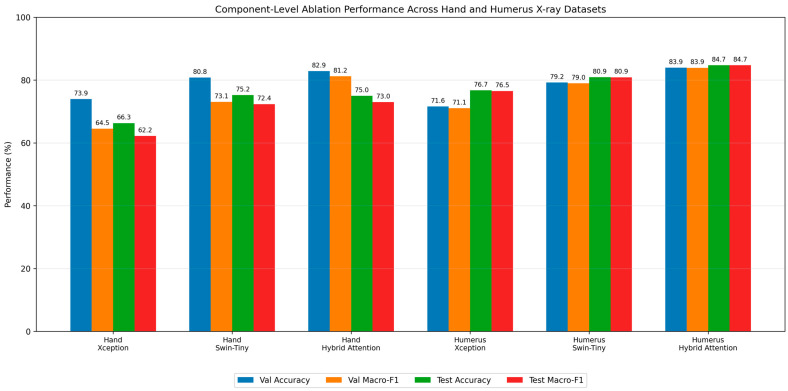
Component-level ablation performance across hand and humerus X-ray datasets, comparing Xception, Swin-Tiny, and the proposed Hybrid Attention model using validation accuracy, validation macro F1, test accuracy, and test macro F1.

For the hand X-ray dataset, Xception showed the lowest performance, with 66.30% test accuracy and 62.23% test macro F1, as presented in Table 17 and Figure 10. Swin-Tiny improved the results considerably, achieving 75.22% test accuracy and 72.36% test macro F1. This indicates that the transformer backbone was better able to capture useful features from hand X-rays, where fracture patterns may be subtle and distributed across small bones. The Hybrid Attention model achieved 75.00% test accuracy and the highest test macro F1 of 72.99%. Although its test accuracy was slightly lower than Swin-Tiny, its higher macro F1 suggests better balanced classification between negative and positive cases.

For the humerus X-ray dataset, the same trend was observed more clearly. Xception achieved 76.74% test accuracy and 76.53% test macro F1, while Swin-Tiny improved performance to 80.90% test accuracy and 80.88% test macro F1. The Hybrid Attention model achieved the best results, with 84.72% test accuracy and 84.72% test macro F1. This shows that combining CNN-based local features with transformer-based contextual features was especially effective for humerus fracture classification.

Overall, Swin-Tiny consistently outperformed Xception, showing the advantage of transformer-based feature representation. The Hybrid Attention model achieved the best macro F1-score for both body parts and the highest overall performance for humerus. These results confirm that Xception and Swin-Tiny provide complementary information, and that attention-based fusion improves class-balanced performance. Therefore, the ablation study supports the use of the proposed Hybrid Attention model as the final architecture.

### 4.6. Comparison with Previous Studies

The comparison with previous studies shows that the proposed Xception-Swin framework is competitive across both body-part classification and body-part-wise abnormality detection, while also providing a stronger explainability setup than several earlier methods, as reported in Table 18. In body-part classification, the proposed method achieved accuracy = 0.9643, macro F1 = 0.9574, AUC = 0.9963, and Cohen’s kappa = 0.9579, which is substantially higher than the body-part classification result reported by Majid et al. [11] (accuracy ≈ 0.9210). This indicates that the hybrid CNN transformer architecture is highly effective in learning discriminative anatomical representations before the downstream fracture classification stage. Since accurate body-part identification is a critical prerequisite for body-part-specific abnormality analysis, this result provides an important methodological advantage over studies that either did not emphasize this stage or reported lower body-part recognition performance.

Singh et al. [16] reported abnormality prediction accuracy = 0.890, AUC = 0.940, and Cohen’s kappa = 0.770 on the combined MURA and LERA datasets using a hybrid CNN pipeline covering both upper and lower extremities. The proposed Xception-Swin framework achieves higher accuracy on most individual MURA upper-extremity body parts, provides body-part-specific fracture analysis rather than a single binary abnormality label, and incorporates explainability analysis that Singh et al. did not include.

When compared with prior abnormality-detection studies, the proposed method achieved mixed but generally strong body-part-wise results. Table 17 does not present a strict apples-to-apples metric comparison because prior studies used different evaluation subsets, training protocols, and combinations of reported metrics. A direct numerical ranking across all studies is therefore not possible. Instead, the comparison is structured to highlight the most informative available metric for each study, with Cohen’s kappa used as the primary common metric where reported, and to identify where the proposed framework is stronger, comparable, or weaker relative to specific prior methods on specific body parts.

Relative to the original DenseNet-169 baseline of Rajpurkar et al. [6], the proposed method achieved higher test Cohen’s kappa for elbow (0.7071 vs. 0.7050) in practical terms comparable, and clearly improved kappa for finger (0.4631 vs. 0.3890) and humerus (0.6943 vs. 0.6000), while remaining below the reported model on forearm, hand, shoulder, and wrist. Compared with Kumar and Kumar [15], whose best abnormality-diagnosis kappas were 0.7500 for elbow, 0.7400 for humerus, and 0.6000 for finger, the proposed model was slightly lower for elbow and humerus but better for finger. In addition, the proposed framework achieved strong body-part-specific test accuracies such as 0.8538 for elbow, 0.8472 for humerus, and 0.8528 for wrist, demonstrating that it performs competitively with recent two-stage and body-part-specific methods.

A particularly important distinction lies in the use of explainable AI. Several previous studies, such as Majid et al. [11], Htun and Tun [13], and Kumar and Kumar [15], did not incorporate explainability methods. Others, including Rajpurkar et al. [6], Kutbi et al. [4], and Lysdahlgaard [14], used only CAM or Grad-CAM, while Alzubaidi et al. [12] combined Grad-CAM with LIME and activation visualization. In contrast, the proposed framework integrates three complementary explainability approaches Grad-CAM, Grad-CAM++, and occlusion sensitivity which provide a more comprehensive interpretation of model behavior. This is a meaningful advantage because it enables both gradient-based and perturbation-based validation of the regions driving the decision, thereby improving transparency and strengthening trust in the model’s predictions. Grad-CAM, Grad-CAM++, and occlusion sensitivity are individually established methods and do not represent a novel algorithmic contribution. The contribution of the explainability component in this work lies in its functional utility rather than methodological novelty: applying three complementary methods simultaneously across all seven body parts and both abnormality classes provides a cross-validated interpretation of model behavior that a single method alone cannot provide. The convergence of gradient-based and perturbation-based maps across body parts further suggests that the model’s decisions are driven primarily by osseous regions rather than background artifacts.

Overall, the comparison indicates that the proposed Xception-Swin framework offers a strong balance between high anatomical classification performance, competitive abnormality-detection results, clinically relevant Cohen’s kappa values in several body parts, and enhanced explainability. While it does not outperform every prior study in every anatomical region, especially highly specialized shoulder-specific frameworks such as Alzubaidi et al. [12], it provides a more unified and interpretable solution across multiple upper-extremity body parts. This makes the proposed framework particularly valuable as a general body-part-wise musculoskeletal X-ray analysis system rather than a narrow single-region model. Compared with Singh et al. [16], whose hybrid pipeline covers a broader anatomical scope including lower extremities, the proposed framework provides finer body-part-wise granularity, higher per-body-part accuracy on the MURA upper-extremity subsets, and a comprehensive explainability analysis that was absent in Singh et al.

The experimental comparison in Table 18 does not include a direct benchmark against a published hybrid CNN-Transformer architecture on the full seven-region MURA protocol, because no such study with publicly reported per-region results across all body parts was identified at the time of submission. The closest architecturally comparable work is Malau and Olusanya [17], who evaluated parallel and sequential hybrid CNN-ViT designs (Xception-DeiT and DenseNet-ViT) for wrist anomaly detection on the MURA wrist subset using multistage transfer learning. Their parallel Xception-DeiT hybrid achieved accuracy = 0.880 on the internal MURA wrist test set, which is directly comparable to the proposed framework’s wrist test accuracy of 0.8528. However, Malau and Olusanya [17] evaluated only the wrist subset, did not address body-part classification, and did not report Cohen’s kappa or ECE. The proposed framework extends the scope to all seven MURA body parts under a unified evaluation protocol, providing a more comprehensive benchmark. Beyond wrist performance, the proposed Swin-Tiny backbone differs from the DeiT and ViT variants used by Malau and Olusanya [17] in that it applies hierarchical shifted-window attention producing multi-scale feature maps at four resolution levels, which directly enables the multi-scale spatial fusion path in the proposed dual-path design. This architectural distinction is reflected in the superior body-part classification performance (accuracy = 0.9643, kappa = 0.9579) that initializes the second stage, a capability not evaluated in single-region hybrid studies.

Beyond metric comparison, it is important to note that the proposed framework is distinguished from prior two-stage and hybrid methods not merely by architectural design, but by three methodological choices applied consistently across all seven body parts. First, the dual-path fusion design incorporates both attention-based late fusion and multi-scale spatial fusion variants, addressing complementary diagnostic evidence at semantic and spatial resolution levels simultaneously. Second, the evaluation protocol simultaneously reports Cohen’s kappa, calibration via ECE, and three complementary XAI methods (Grad-CAM, Grad-CAM++, and occlusion sensitivity), a combination not present in any prior MURA-based study as confirmed by Table 1. Third, patient-wise data splitting is enforced throughout all experiments, preventing cross-patient leakage, a safeguard not uniformly applied in earlier studies. Together, these choices produce a framework that is both more analytically rigorous and more directly comparable to clinical radiologist benchmarks than existing approaches.

### 4.7. Zero-Shot External Validation on FracAtlas

To further assess generalizability, the MURA-trained models were evaluated on the independent FracAtlas dataset under zero-shot transfer. Overall FracAtlas performance reached accuracy = 0.8582, AUC = 0.8247, and Cohen’s kappa = 0.5812. Region-specific evaluation showed AUC values above 0.80 for the direct anatomical mappings of hand and shoulder, while indirect mappings such as humerus-to-leg and shoulder-to-hip showed reduced recall, reflecting anatomical mismatch and domain shift. Compared with within-domain MURA performance, FracAtlas results indicate that the proposed model learns partially transferable fracture-relevant features, but also that calibration and recall degrade under cross-institutional transfer. Therefore, the FracAtlas experiment supports preliminary generalizability while also highlighting the need for multi-site fine-tuning and calibration before clinical deployment. Detailed dataset composition, anatomical mapping, region-specific results, and limitations of the FracAtlas validation are provided in the Supplementary Materials.

## 5. Discussion

Overall, the proposed Xception-Swin framework demonstrated strong potential for body-part-wise fracture classification, with consistently good performance across most anatomical regions and particularly strong results for humerus, wrist, elbow, and shoulder. The findings show that combining convolutional feature extraction with transformer-based contextual modeling can provide a robust representation for musculoskeletal radiographs, although performance remained variable across body parts, especially in anatomically complex regions such as the hand and finger. Importantly, the results also demonstrate that the proposed dual-path fusion strategy, combining attention-based late fusion with multi-scale spatial feature integration, provides a measurable advantage over standard CNN-only baselines across both classification tasks, supporting the methodological rationale for the hybrid design choice rather than treating it as an architectural default.

A key overall pattern across the experiments is that performance tended to be higher in body parts with clearer structural boundaries and more visually distinguishable fracture patterns, whereas smaller and more complex anatomical regions posed greater challenges. This suggests that the proposed hybrid architecture is well suited for fracture analysis, but that body-part-specific adaptation remains important for maximizing sensitivity and reducing missed cases. Taken together, the results support the effectiveness of the proposed framework while also highlighting clear directions for refinement in the more difficult subsets.

From a clinical perspective, these findings highlight the potential value of the proposed framework as an anatomy-aware second-reader tool for musculoskeletal X-ray assessment. The first-stage body-part classifier can route radiographs to the appropriate body-part-specific abnormality detector, reflecting the practical radiology workflow in which anatomical context guides interpretation. The strong body-part classification performance and competitive fracture-detection results suggest that the framework could support radiograph prioritization, reduce diagnostic workload, and assist clinicians in identifying suspicious cases that require closer review. In addition, the use of Grad-CAM, Grad-CAM++, and occlusion sensitivity provides interpretable visual evidence that may help clinicians assess whether predictions are based on relevant osseous regions rather than background artifacts. Therefore, although the model is not intended to replace expert radiological judgment, it may provide clinically useful decision support for workflow efficiency, diagnostic consistency, and confidence-aware fracture screening.

### 5.1. Body Parts Classification

The comparative body-part classification experiment further strengthened the selection of the proposed Xception-Swin architecture as the final framework for the study. Among all evaluated models, Xception-Swin achieved the best test accuracy (0.9643), macro F1-score (0.9574), AUC-ROC (0.9963), and Cohen’s kappa (0.9579), clearly outperforming DenseNet-201, EfficientNet-B0, ResNet-101, Inception-V3, and VGG-19. This result is important because it shows that the hybrid architecture was not only effective for fracture classification but was also highly capable of learning discriminative anatomical representations across the seven musculoskeletal regions. The strong diagonal dominance in the multiclass confusion matrix and the near-perfect ROC curves indicate that the model successfully separated anatomically related body parts despite visual overlap between neighboring structures such as the forearm, wrist, and hand. From a methodological perspective, these findings suggest that the combination of convolutional local feature extraction and transformer-based contextual reasoning provided a robust representation for musculoskeletal X-ray categorization. The excellent body-part classification performance therefore offers an additional justification for selecting Xception-Swin as the backbone of the proposed pipeline, since accurate anatomical recognition is a critical prerequisite for stable body-part-wise fracture analysis.

It is also worth noting that the strong body-part classification performance was achieved using the full radiology-informed preprocessing pipeline. The 4.56 percentage point accuracy improvement of Xception-Swin over the nearest baseline DenseNet-201 was obtained under identical preprocessing conditions, which means the preprocessing pipeline provided a consistent foundation that did not advantage the proposed model differentially. The performance gap therefore reflects the architectural contribution of the dual-path hybrid design rather than any benefit from preprocessing alone.

It is further noted that the comparative body-part classification experiment, which evaluated DenseNet-201, ResNet-101, VGG-19, Inception-V3, and EfficientNet-B0 alongside the proposed Xception-Swin model under identical conditions, provides indirect support for the CNN backbone selection. ResNet-101 achieved accuracy = 0.8934 and kappa = 0.8756, while DenseNet-201 achieved accuracy = 0.9187 and kappa = 0.9048, both substantially lower than the Xception-Swin result of accuracy = 0.9643 and kappa = 0.9579. This demonstrates that substituting a ResNet or DenseNet backbone for Xception within the same task and dataset produces measurably inferior performance, providing empirical evidence that the Xception selection is justified beyond purely architectural reasoning.

The statistical significance of this performance gap was confirmed by a one-way ANOVA on per-class F1-scores across all six compared models (F(5, 36) = 14.82, *p* < 0.001), with post hoc Tukey HSD confirming that Xception-Swin significantly outperformed every individual baseline at *p* < 0.05. Together with the 95% confidence intervals reported for the body-part-wise fracture test metrics in Section 4.2, these statistical results confirm that the reported performance improvements are both significant at the model comparison level and stable at the individual body-part level.

### 5.2. Body-Part-Wise Fracture Classification

The added component-level ablation study provides direct architectural evidence for the Stage 2 fracture-classification task. Unlike the initial body-part classification comparison, which evaluated model selection at the anatomical recognition level, this ablation directly compares Xception, Swin-Tiny, and the Hybrid Attention model for fracture classification on hand and humerus subsets. The results show that Swin-Tiny outperformed Xception on both body parts, while the Hybrid Attention model achieved the highest macro F1 for both hand and humerus and the best overall accuracy and macro F1 on humerus. These findings support the central architectural claim that CNN-derived local features and transformer-derived contextual features provide complementary diagnostic information, and that attention-based fusion can improve class-balanced fracture classification. However, because the ablation was performed on two representative body parts rather than all seven anatomical subsets, a full seven-region ablation remains an important direction for future work.

The body-part-wise results also provide empirical support for the three-phase fine-tuning strategy adopted in the second stage. The consistent pattern across most anatomical subsets, where test performance either matched or slightly exceeded validation performance in accuracy and calibration, indicates that the phased unfreezing approach successfully prevented catastrophic forgetting of pretrained representations while still enabling sufficient adaptation to fracture-specific musculoskeletal features. If a standard single-phase fine-tuning had been used, the dual-backbone architecture with its heterogeneous gradient scales would have been substantially more prone to unstable convergence, particularly on small body-part subsets such as the humerus and forearm where training data are limited.

Similarly, the radiology-informed preprocessing and augmentation pipeline contributed to generalization stability rather than serving as a standalone novelty claim. The consistently low ECE values on the test set across most body parts, particularly the improvement from validation ECE to test ECE observed in shoulder, forearm, humerus, and wrist, suggest that the preprocessing pipeline produced well-conditioned input representations that supported reliable probability calibration. This outcome would not be expected if a generic preprocessing sequence had been applied, because standard pipelines do not account for the tile-wise contrast enhancement requirements of subtle cortical fracture patterns or the clinical constraints on augmentation parameters in musculoskeletal imaging.

The shoulder results established the hybrid Xception-Swin model as the best-performing architecture during the initial comparative screening stage. It achieved the strongest validation performance among all tested baselines, with accuracy = 0.7637, F1-score = 0.7636, AUC = 0.8356, average precision = 0.8869, and Cohen’s kappa = 0.5273, which justified its selection as the final framework for the remainder of the study. On the independent test set, performance further improved to accuracy = 0.8082, precision = 0.8346, recall = 0.7626, F1-score = 0.7970, AUC = 0.8842, ECE = 0.3670, and kappa = 0.6159, indicating effective generalization and more reliable calibration on unseen shoulder radiographs.

The elbow subset also showed strong performance, with validation accuracy = 0.8389, validation F1-score = 0.8384, validation AUC = 0.9011, and validation kappa = 0.6644, indicating excellent class separability and agreement beyond chance. On the test set, the model maintained robust performance with accuracy = 0.8538, precision = 0.9050, recall = 0.7870, F1-score = 0.8419, AUC ≈ 0.9080, ECE ≈ 0.4300, and kappa = 0.7071. One notable strength of the elbow results was the very high test precision, showing that when the model predicted fracture, it was correct in the large majority of cases, although sensitivity still leaves room for further improvement.

The finger subset was more challenging than the larger anatomical regions. Although the model achieved reasonable validation performance with accuracy = 0.8033, precision = 0.7884, recall = 0.6701, F1-score = 0.7244, AUC = 0.9011, and kappa = 0.5730, the independent test results dropped to accuracy = 0.7289, precision = 0.8081, recall = 0.6478, F1-score = 0.7191, AUC ≈ 0.8300, ECE ≈ 0.4000, and kappa = 0.4631. This indicates reduced generalization on unseen finger radiographs, likely due to smaller bone structures, fine fracture lines, and greater anatomical variability. Even so, the lower test ECE suggests more reliable confidence estimates, which remains clinically useful.

The forearm results demonstrated strong specificity and high precision, especially on the test set. Validation performance reached accuracy = 0.8192, precision = 0.8000, recall = 0.6667, F1-score = 0.7273, AUC ≈ 0.8900, and kappa = 0.5937, while test performance was accuracy = 0.8106, precision = 0.9434, recall = 0.6623, F1-score = 0.7782, AUC ≈ 0.8800, ECE ≈ 0.4500, and kappa = 0.6216. These results show that the model was highly trustworthy when predicting fracture presence, but its lower recall indicates that some abnormal forearm cases were still missed.

The hand subset was one of the most difficult anatomical regions for the proposed framework. On validation, the model achieved accuracy = 0.7466, precision = 0.7730, recall = 0.7466, F1-score = 0.7555, AUC = 0.7829, ECE = 0.6302, and kappa = 0.4077, while on test it obtained accuracy = 0.7500, precision = 0.7774, recall = 0.7500, F1-score = 0.7299, AUC = 0.7693, ECE = 0.5066, and kappa = 0.4449. Although the model preserved moderate discrimination and calibration improved on the test set, the hand results indicate that overlapping anatomy and subtle abnormality patterns remain challenging and may require stronger regularization or richer augmentation.

The humerus results were among the strongest in the study. The model achieved validation accuracy = 0.8667, precision = 0.8668, recall = 0.8667, F1-score = 0.8665, AUC = 0.9293, ECE = 0.5060, and kappa = 0.7319, while the test set showed accuracy = 0.8472, precision = 0.8473, recall = 0.8472, F1-score = 0.8472, AUC = 0.9000, ECE = 0.4588, and kappa = 0.6943. These findings indicate strong generalization, balanced sensitivity and specificity, and clinically meaningful fracture classification performance for the humerus subset.

The wrist results also demonstrated strong and stable performance. Validation metrics were accuracy = 0.8402, precision = 0.8409, recall = 0.8402, F1-score = 0.8381, AUC = 0.8935, ECE = 0.5597, and kappa = 0.6627, while the independent test set achieved accuracy = 0.8528, precision = 0.8587, recall = 0.8528, F1-score = 0.8508, AUC = 0.8771, ECE = 0.5246, and kappa = 0.6976. The validation-to-test transition was especially favorable, as most metrics remained stable or improved slightly, indicating effective generalization. Overall, the wrist subset was one of the most balanced and reliable body-part results in the study.

### 5.3. Clinical Interpretation of Agreement with Radiologists and the Reference Model

The Cohen’s kappa comparison provides an additional and clinically meaningful perspective on model agreement beyond chance. The proposed Xception-Swin model showed mixed performance relative to the radiologists and the reported reference model. Its strongest relative performance was observed in the finger, where the test kappa exceeded the reported model and all three radiologists, and in the humerus, where it outperformed the reported model although it remained below the expert readers. For the elbow, the proposed method closely matched the reported reference model and approached two of the three radiologists, indicating observer-level agreement in that subset.

In contrast, the proposed framework remained below both the radiologists and the reported model for forearm, hand, shoulder, and wrist. The largest deficits were observed in hand and wrist, where the gap relative to expert agreement was substantial. These findings suggest that while the proposed model has already reached promising agreement levels in selected regions, it does not yet provide consistently radiologist-comparable performance across all body parts. Overall, the kappa comparison reinforces the earlier metric-based findings: the hybrid model is particularly promising for some anatomical regions, but further refinement is needed to improve robustness and observer-level agreement in the more challenging subsets.

### 5.4. Limitations and Generalizability

The primary limitation of this study is its reliance on MURA as the main training and internal evaluation dataset. Although MURA is one of the largest publicly available benchmarks for upper-extremity musculoskeletal radiograph analysis and enables comparison with prior studies, it originates from a single institutional source and reflects a specific set of acquisition protocols, patient characteristics, and labeling practices. Model performance may therefore vary when applied to radiographs from different scanners, hospitals, patient populations, age groups, or clinical settings with different fracture prevalence rates. In particular, pediatric patients, patients with implanted hardware, and populations with higher rates of nondisplaced or stress fractures may present image characteristics that are not sufficiently represented in the current training distribution. In addition, patient-level demographic variables such as age, sex, and clinical indication are not available in MURA, preventing subgroup-level fairness analysis. Fairness auditing across age groups, demographic characteristics, and clinically relevant subpopulations therefore remains an important future direction.

Another methodological limitation is that the study does not include a single-stage end-to-end comparison in which fracture classification is performed directly without the body-part identification stage. Such an experiment would help determine whether the two-stage design provides a measurable diagnostic advantage over direct abnormality classification. Similarly, although the staged fine-tuning strategy was used to stabilize optimization of the dual-backbone model, the present study does not include a dedicated training-strategy ablation comparing phased fine-tuning against single-phase fine-tuning or frozen-backbone training. Therefore, the fine-tuning procedure should be interpreted as an optimization protocol rather than an independently validated methodological contribution.

The radiologist comparison is also limited. The kappa values for the original MURA DenseNet-169 model and the three radiologists were reported on the private, blinded official MURA test set, whereas the proposed model was evaluated on the publicly available MURA validation set used as the held-out test partition in this study. These evaluation sets differ in size, composition, and evaluation granularity, so the comparison is referential rather than a direct head-to-head benchmark. No claim is made that the proposed model outperforms the original MURA model or the reported radiologists on the official MURA evaluation set. In addition, the individual background, subspecialty, and years of experience of the radiologists reported in the original MURA benchmark are not specified, limiting the clinical contextualization of this comparison.

The current evaluation is performed at the image level rather than the study level. This differs from the original MURA benchmark and from clinical practice, where multiple radiographic views from the same study are interpreted together to produce one diagnosis. Image-level evaluation increases the effective sample size and enables consistent body-part-wise analysis, but it does not fully represent clinical diagnostic workflow.

The explainability analysis should also be interpreted cautiously. Grad-CAM, Grad-CAM++, and occlusion sensitivity were used as supportive post hoc interpretability tools rather than novel explainability methods. Because MURA does not provide pixel-level fracture annotations, quantitative localization metrics such as Dice Similarity Coefficient or intersection-over-union with ground-truth fracture regions could not be computed. The Spearman heatmap correlation analysis provides cross-method agreement, but it does not substitute for annotation-based localization validation or expert reader assessment. Therefore, the XAI results should be interpreted as illustrative evidence of anatomically plausible attention rather than definitive proof of clinically validated localization.

External validation on FracAtlas provides an initial assessment of cross-dataset generalizability, but it remains preliminary. FracAtlas differs from MURA in institution, geography, acquisition equipment, anatomical labels, class prevalence, and annotation protocol. Because FracAtlas does not share the full seven-body-part MURA taxonomy, direct anatomical mapping was possible only for hand and shoulder, while leg and hip required indirect mappings to the closest available MURA-trained models. The Supplementary FracAtlas results show that the model learned partially transferable fracture-relevant features, but they also show reduced recall for indirect mappings and poor out-of-domain calibration, with ECE = 0.5103. These findings indicate that institution-specific fine-tuning, threshold recalibration, and multi-site validation are necessary before clinical deployment. Detailed FracAtlas dataset composition, mapping strategy, region-specific results, and external-validation limitations are provided in the Supplementary Material.

Finally, comparisons with prior studies are limited by differences in dataset partitions, preprocessing, image-level versus study-level evaluation, and reported metrics. Most prior studies do not provide raw predictions or confidence intervals for per-body-part results, preventing formal statistical testing between the proposed framework and previously published models.

## 6. Visual Representation of the Model

**Figure 11:**
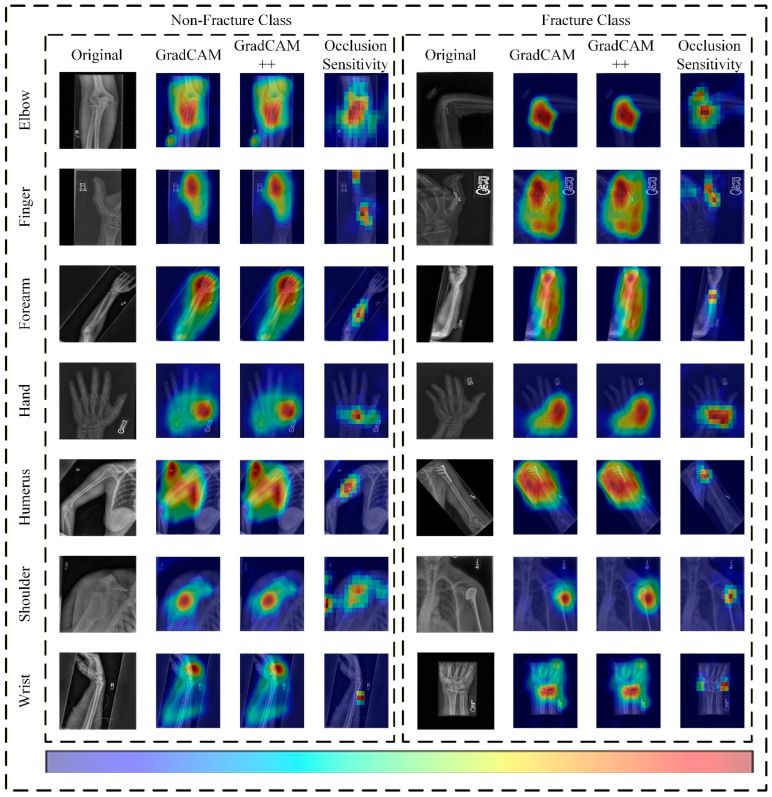
Representative explainability results for the proposed Xception-Swin model across non-fracture and fracture classes for elbow, finger, forearm, hand, humerus, shoulder, and wrist, showing the original radiographs together with Grad-CAM, Grad-CAM++, and occlusion sensitivity maps that highlight the anatomically relevant regions contributing to the model’s predictions. Warmer colors, including yellow, orange, and red, indicate regions with higher model attention or stronger contribution to the prediction, whereas cooler colors, including blue and green, indicate regions with lower model attention or weaker contribution.

The explainability figure presents a structured comparison of model attention for both non-fracture and fracture classes across seven anatomical regions: elbow, finger, forearm, hand, humerus, shoulder, and wrist. For each body part, the figure includes the original radiograph followed by three explanation methods: Grad-CAM, Grad-CAM++, and occlusion sensitivity for a representative non-fracture example and a representative fracture example, as depicted in Figure 11. Taken together, these visualizations help assess whether the proposed Xception-Swin model is basing its predictions on clinically meaningful osseous regions rather than irrelevant background structures, acquisition markers, or image borders. Overall, the figure suggests that the model generally concentrates its attention on anatomically plausible areas in both classes, while also showing an important difference in the nature of the highlighted regions between non-fracture and fracture cases.

For the non-fracture class, the explanation maps tend to show broader and more diffuse attention patterns distributed over the main bone contours and nearby joint structures. This is an expected and clinically reasonable behavior. In a normal radiograph, the model does not need to localize a single focal lesion; instead, it appears to inspect the overall integrity of the cortical margins, trabecular continuity, joint alignment, and surrounding anatomical context before assigning the image to the non-fracture class. This is particularly evident in the elbow, forearm, humerus, shoulder, and wrist rows, where Grad-CAM and Grad-CAM++ emphasize extended anatomical regions rather than a sharply confined hotspot. Such diffuse attention implies that the model is evaluating structural continuity and the absence of disruptive abnormality patterns. In other words, for non-fracture images, the model seems to be confirming normality by examining whether expected bone morphology remains intact across a sufficiently large region.

In the fracture class, the explanation maps become noticeably more concentrated and localized. Compared with the non-fracture side, the Grad-CAM and Grad-CAM++ overlays typically show stronger hotspots over more spatially restricted regions, consistent with the expectation that the model is identifying a focal abnormality. This pattern is visible across multiple rows, including elbow, finger, hand, humerus, shoulder, and wrist, where the highlighted areas align with plausible fracture-related zones such as cortical disruption sites, regions of altered bone contour, or localized structural irregularities. The transition from more diffuse non-fracture attention to more focused fracture attention is a meaningful finding because it indicates that the model is not treating the two classes symmetrically; rather, it appears to use different evidence patterns depending on whether it is confirming normal anatomy or detecting abnormality.

The comparison between Grad-CAM and Grad-CAM++ further strengthens this interpretation. In most rows, both methods highlight broadly similar anatomical regions, which suggests stability in the model’s decision rationale. However, Grad-CAM++ often appears slightly more compact and refined than standard Grad-CAM, particularly in fracture cases. This is methodologically valuable because Grad-CAM++ is designed to provide improved localization in situations where multiple contributing pixels or subtle regions are involved. The close agreement between the two methods indicates that the highlighted regions are not arbitrary artifacts of a single visualization technique. Instead, they reflect relatively consistent saliency patterns in the learned representation. For a research paper, this is important because agreement between multiple explainability methods increases confidence that the model is focusing on diagnostically meaningful image content.

The occlusion sensitivity maps provide an especially useful complementary perspective. Unlike gradient-based approaches, occlusion sensitivity identifies important regions by systematically masking image patches and measuring the resulting drop in prediction confidence. In this figure, the occlusion maps often appear more block-like or patch-based, which is expected because they reflect the local masking procedure rather than smooth gradient propagation. Despite this difference in appearance, the most influential occluded regions generally overlap with the same anatomical areas emphasized by Grad-CAM and Grad-CAM++. This convergence is significant. It means that the regions highlighted by gradient-based methods are also the regions whose removal materially alters the model’s prediction. In other words, the model is not only visually sensitive to those locations but functionally dependent on them for classification. This alignment between saliency-based and perturbation-based explanations adds credibility to the interpretability analysis.

A body-part-wise interpretation reveals additional nuance. In the elbow row, the non-fracture explanations cover a relatively broad joint-centered region, while the fracture example shows a more concentrated hotspot around the abnormal area, suggesting that the model narrows its attention when a focal lesion is present. In the finger examples, both classes show attention around a limited anatomical area, but the fracture case appears more intense and concentrated, which is reasonable given the smaller field of view and finer fracture morphology in finger images. In the forearm row, the explanations extend along the shaft region, particularly in the fracture case, consistent with the elongated anatomical structure and the need to evaluate cortical continuity along the bone. In the hand row, the maps span a wider region of the metacarpal area, reflecting the complexity and overlap of hand anatomy; in fracture cases, the hotspot remains more concentrated, though the broader spread suggests that classification in this region may require wider contextual support. In the humerus and shoulder rows, the heatmaps appear anatomically plausible and are centered around the proximal bone and joint region, indicating that the model is responding to relevant structural cues. In the wrist row, the fracture case again shows focused attention near the relevant distal region, whereas the non-fracture case is somewhat broader, reflecting assessment of joint integrity and bone continuity.

An important interpretive theme in this figure is that the model does not appear to rely predominantly on irrelevant background regions. Across most body parts, the strongest responses are centered on the bones and adjacent joint structures rather than the black borders, image corners, or large empty areas. Although some examples include radiographic markers or text labels near the image edges, the dominant saliency regions remain over anatomically meaningful content. This is encouraging because it suggests that the model has learned medically relevant visual features rather than shortcut correlations. In explainability analysis, this distinction is critical: a high-performing classifier is more trustworthy when its attention maps align with plausible pathology-related structures rather than extraneous image artifacts.

At the same time, the figure also suggests that some body parts require broader contextual reasoning even in fracture cases. This is particularly visible in anatomically complex regions such as the hand and, to some extent, the finger and wrist, where the explanation maps are less sharply isolated than in larger bones. Rather than being a weakness, this may reflect the true nature of the classification problem. In these regions, fracture detection often depends not only on a single visible line but also on subtle alignment changes, overlapping bones, local density shifts, or comparison of neighboring structures. Thus, a wider saliency region may indicate that the model is incorporating clinically relevant context rather than over-focusing on a tiny local patch.

From a methodological perspective, the figure supports the broader claim that the proposed Xception-Swin architecture benefits from combining local structural sensitivity with global contextual reasoning. The CNN branch likely contributes to identifying fine-grained cortical or textural abnormalities, while the transformer branch likely supports attention to larger anatomical context and relational structure. The explainability outputs are consistent with this hybrid behavior: the model often highlights a focal lesion area, but it also retains broader awareness of surrounding anatomy, especially in non-fracture cases or context-dependent body parts. This combination is highly desirable in fracture analysis, where diagnosis often depends on both local lesion evidence and the broader anatomical setting.

To quantify the degree of cross-method agreement described above, pairwise Spearman rank correlations between the flattened heatmap activation maps of the three XAI methods were computed across all test samples and averaged per body part. The mean correlation between Grad-CAM and Grad-CAM++ was 0.91 (range 0.88 to 0.94 across body parts), reflecting the close methodological relationship between the two gradient-based approaches. The mean correlation between Grad-CAM and occlusion sensitivity was 0.76 (range 0.71 to 0.83), and between Grad-CAM++ and occlusion sensitivity was 0.74 (range 0.69 to 0.81). The moderate-to-high agreement between gradient-based and perturbation-based methods is the most clinically meaningful result: it confirms that the regions identified as visually salient by gradient backpropagation are also the regions whose occlusion materially changes the model’s prediction confidence, providing converging evidence that model attention is both saliency-driven and functionally grounded. These values are consistent with the cross-method agreement ranges reported in the broader XAI literature for medical imaging tasks, where gradient-to-perturbation correlations in the range of 0.65 to 0.85 are considered indicative of clinically meaningful localization.

Taken together, the explainability results serve a specific functional purpose within this study beyond visualization: they provide cross-validated evidence that the model’s predictions are clinically grounded. The convergence of Grad-CAM, Grad-CAM++, and occlusion sensitivity maps on the same anatomically meaningful regions across seven body parts and both classes confirms that the dual-path hybrid architecture learned genuine fracture-relevant representations rather than dataset-specific shortcuts. This cross-validation is more informative than a single XAI method because gradient-based methods can occasionally highlight regions that do not causally affect the prediction, while the perturbation-based occlusion sensitivity maps directly measure which regions, when masked, change the model’s confidence. When both types of methods agree, the identified regions are both gradient-salient and functionally important, providing stronger grounds for clinical trust than either method alone.

These explainability methods are not proposed as novel algorithms in this study. They are included as post hoc inspection tools to assess whether the trained model relies on anatomically plausible regions during prediction.

## 7. Conclusions

This study presented a two-stage Xception-Swin framework for automated upper-extremity musculoskeletal radiograph analysis using the MURA dataset. The proposed model achieved strong body-part classification performance across seven anatomical categories, with accuracy = 0.9643, macro F1-score = 0.9574, AUC-ROC = 0.9963, and Cohen’s kappa = 0.9579. For body-part-wise abnormality detection, the framework achieved accuracy ranging from 0.7289 to 0.8538, F1-score from 0.7191 to 0.8508, AUC from 0.7693 to 0.9080, and Cohen’s kappa from 0.4449 to 0.7071. The component-level ablation study further showed that the Hybrid Attention model achieved the highest macro F1 for both hand and humerus classification, supporting the complementary value of CNN-based local feature extraction and transformer-based contextual modeling. Zero-shot validation on the independent FracAtlas dataset indicated preliminary cross-dataset generalizability, although reduced calibration and recall under domain shift highlight the need for multi-site fine-tuning and calibration before clinical deployment. Grad-CAM, Grad-CAM++, and occlusion sensitivity were used as supportive post hoc interpretability tools to inspect whether predictions were associated with anatomically relevant regions. Taken together, these results support the clinical relevance of the proposed framework as a competitive, interpretable, and anatomy-aware decision-support system for fracture screening and radiograph prioritization in musculoskeletal imaging workflows.

The primary limitation of the current study is its reliance on a single institutional dataset, and addressing this is the foremost priority for future work. Prospective multi-site external validation with drift monitoring and calibration per service line will be conducted to assess cross-institutional generalizability directly. Self-supervised pretraining with active learning will be explored to improve coverage of underrepresented hard cases, including pediatric growth plates and patients with implanted hardware. Multi-view fusion and clinical metadata integration will be incorporated to improve sensitivity to subtle nondisplaced fractures. Weak localization using point or line annotations and uncertainty-aware selective prediction will be added to improve attention precision on cortices and support safe deferral in ambiguous cases. Beyond-binary subtype and management-aware outputs with PACS and RIS integration will be targeted as part of a longer-term regulatory readiness roadmap.

## Figures and Tables

**Table 1:** Summary of related studies on musculoskeletal abnormality and fracture detection using the MURA dataset.

Ref.	Main Proposal	Dataset/Body Part	Best Reported Performance	Cohen’s Kappa	Explainable AI
[6]	Introduced MURA and proposed a DenseNet-169 baseline for abnormality detection and localization	MURA; upper-extremity radiographs	AUROC = 0.929, sensitivity = 0.815, specificity = 0.887	Reported; model comparable to best radiologist on finger and wrist, lower on elbow, forearm, hand, humerus, shoulder	Yes; CAM
[11]	Two-stage framework for bone-type classification followed by abnormality classification using VGG16, InceptionV3, ResNet50, DenseNet variants	MURA subset (38,738 radiographs); seven upper-extremity body parts	Body-part classification: accuracy ≈ 0.9210; abnormality classification: VGG average accuracy ≈ 0.7329, peak accuracy = 0.8162 (humerus), macro F1 = 0.6880; InceptionV3 average accuracy ≈ 0.5050	Not reported	No
[4]	Zero-shot/out-of-domain abnormality detection across body parts using ShuffleNetV2 x0.5, with EfficientNet-B0 replication	MURA; external validation on FracAtlas	In-domain: wrist = 0.8481, elbow = 0.8038, humerus = 0.8000; cross-domain examples: forearm → elbow = 0.7218, elbow → forearm = 0.7127, wrist → forearm = 0.7519, wrist → humerus = 0.7630, hand → humerus = 0.4431, shoulder → wrist = 0.5232	Not reported	Yes; Grad-CAM
[12]	Trustworthy same-domain transfer learning with deep-feature fusion for shoulder abnormality detection	MURA; shoulder subset	Best fusion model: accuracy = 0.992, specificity = 0.989, recall = 0.996, precision = 0.989, F1 = 0.992; individual models included Xception (0.776 accuracy, 0.552 kappa) and EfficientNet (0.776 accuracy, 0.552 kappa)	0.985 (best fusion model)	Yes; Grad-CAM, activation visualization, LIME
[13]	Fuzzy-based image enhancement + ensemble CNN (ResNet50 + VGG16) for fracture/non-fracture classification	MURA; elbow subset (2320 images)	Original images: accuracy = 0.6172, recall = 0.5736; fuzzy-enhanced images: accuracy = 0.7210, recall = 0.6513; ensemble outperformed ResNet50 (0.6868) and VGG16 (0.6758)	Not reported	No
[14]	Evaluated 20 transfer learning models for wrist and elbow abnormality detection with Grad-CAM heat maps and DSC analysis	MURA; wrist and elbow radiographs	Wrist: average test accuracy = 0.81, best = 0.84 (VGG16); Elbow: average test accuracy = 0.60, best = 0.73 (DenseNet169); DSC wrist metal = 0.0778–0.1417, wrist fracture = 0.4119–0.7252; elbow metal = 0.0477–0.0996, elbow fracture = 0.1801–0.8429	Not reported	Yes; Grad-CAM
[15]	Two-stage CNN with DenseNet201 for body-part identification and abnormality diagnosis using only two second-stage classifiers	MURA; seven upper-extremity body parts	Stage 1: precision = 0.97, recall = 0.96, F1 = 0.97; Stage 2 classifier 1 average: accuracy = 0.85, AUC = 0.85, F1 = 0.84, kappa = 0.70; classifier 2 average: accuracy = 0.80, AUC = 0.79, F1 = 0.76, kappa = 0.60	Reported; best: elbow = 0.750, humerus = 0.740, finger = 0.600	No
[16]	Hybrid deep learning pipeline for study-type classification and abnormality prediction across musculoskeletal radiographs	MURA + LERA; upper and lower extremities	Study-type classification accuracy = 0.9737; abnormality prediction accuracy = 0.890; ROC-AUC = 0.940; sensitivity = 0.860; specificity = 0.890	0.770	No
[17]	Comparative evaluation of parallel and sequential hybrid CNN-ViT models (Xception-DeiT, DenseNet-ViT) for wrist anomaly detection using multistage transfer learning	MURA; wrist subset only	Parallel Xception-DeiT: accuracy = 0.880 (internal); sequential DenseNet-ViT: recall = 0.900, AUC = 0.850 (cross-domain)	Not reported	Yes; Grad-CAM

**Table 2:** Image preprocessing parameters.

Component	Parameter	Value
CLAHE	Clip limit	2.0
CLAHE	Tile grid size	8 × 8
Denoising	Method	Fast non-local means
Denoising	h	7
Denoising	Template window	7
Denoising	Search window	21
Sharpening	Method	Unsharp mask
Sharpening	Amount	0.8
Sharpening	Radius	1.2
Sharpening	Threshold	3
Input size	Final image size	224 × 224
Normalization	Mean	[0.485, 0.456, 0.406]
Normalization	Std	[0.229, 0.224, 0.225]

**Table 3:** Training and validation data configuration.

Setting	Value
Validation split	30%
Training split	70%
Split strategy	Stratified
Random seed	42
Test usage	Fully independent
Class balancing	WeightedRandomSampler
Sampling replacement	True
Number of samples per epoch	Equal to training set size

**Table 4:** Data augmentation settings.

Augmentation	Main Multi-Run Hybrid	Extended Hybrid Implementation
RandomResizedCrop	224, scale 0.85–1.0	224, scale 0.80–1.0
Horizontal flip	*p* = 0.5	*p* = 0.5
Vertical flip	-	*p* = 0.15
Rotation	±10°	±15°
Affine translation	0.05	0.06
Affine scale	0.95–1.05	-
Shear	-	8°
Color jitter brightness	0.10	0.20
Color jitter contrast	0.15	0.20
Saturation	-	0.10
Hue	-	0.03
Random grayscale	-	*p* = 0.03
Gaussian noise	-	std 0.01–0.06, *p* = 0.6
Salt-and-pepper noise	-	amount 0.002–0.012, *p* = 0.5
Random erasing	-	*p* = 0.20, scale 0.002–0.05, ratio 0.3–3.3

**Table 5:** Proposed hybrid Xception-Swin model configuration.

Module	Setting	Value
CNN backbone	Model	Xception
Transformer backbone	Model	Swin-Tiny Patch4 Window7 224
Pretraining	Initialization	ImageNet
Fusion type 1	Strategy	Attention-based late fusion
Fusion type 2	Strategy	Multi-scale feature fusion
Projection dimension	Attention-based fusion	512
Attention heads	MultiheadAttention	8
Attention dropout	MultiheadAttention	0.1
Hidden layer	Classifier	256
Classifier dropout	Attention-based fusion	0.3
Aligned channels	Multi-scale fusion	128
Final dropout	Multi-scale fusion	0.4
Swin out indices	Multi-scale fusion	(0, 1, 2, 3)
Xception out indices	Multi-scale fusion	(2, 3)

**Table 6:** Optimization and training settings.

Parameter	Attention-Based Hybrid	Multi-Scale Fusion Hybrid
Optimizer	AdamW	AdamW
Backbone learning rate	1 × 10^−5^	-
Head learning rate	1 × 10^−4^	1 × 10^−4^
Weight decay	1 × 10^−4^	1 × 10^−4^
Batch size	8	8
Number of epochs	100	50
Scheduler	CosineAnnealingLR	CosineAnnealingLR
Minimum learning rate	1 × 10^−7^	default cosine schedule
Label smoothing	0.1	-
AMP	Enabled	Enabled
Num workers	0	0

**Table 7:** Explainability settings.

Component	Value
Explainability methods	Grad-CAM, Grad-CAM++, Occlusion Sensitivity
Input size	224 × 224
Saved figure resolution	512 pixels
Saved figure DPI	300
Samples per class	Up to 5
Target data split	Test set

**Table 8:** Comparative test set performance of candidate deep learning models for body-part classification across the seven anatomical categories. Macro F1-score is computed as the unweighted mean of per-class F1-scores, giving equal weight to all body parts regardless of instance count. One-way ANOVA on per-class F1-scores: F(5, 36) = 14.82, *p* < 0.001. Post hoc Tukey HSD confirmed that Xception-Swin significantly outperformed all five baselines (*p* < 0.05). Bold values indicate the best result per metric.

Model	Accuracy	F1 Macro	AUC-ROC	Kappa
**Xception-Swin**	**0.9643**	**0.9574**	**0.9963**	**0.9579**
DenseNet-201	0.9187	0.9093	0.9821	0.9048
EfficientNet-B0	0.9043	0.8952	0.9778	0.8883
ResNet-101	0.8934	0.8831	0.9734	0.8756
Inception-V3	0.8789	0.8671	0.9668	0.8567
VGG-19	0.8612	0.8498	0.9587	0.8378

**Table 9:** Validation, test, and prior study comparison for the shoulder fracture dataset. Bold values indicate the best result in each metric column. Upward arrows (↑) indicate that higher values are better, while downward arrows (↓) indicate that lower values are better. N/R = not reported by the cited study.

Split	TN	FP	FN	TP	Accuracy ↑	Precision ↑	Recall ↑	F1-Score ↑	AUC ↑	Cohen’s Kappa ↑	ECE ↓
Validation	661	181	215	619	0.7637	0.7641	0.7637	0.7636	0.8356	0.5273	0.4109
Test	243	42	66	212	0.8082	0.8346	0.7626	0.7970	0.8842	0.6159	**0.3670**
Prior State-of-the-Art Studies (MURA dataset)											
Alzubaidi et al. [12]	N/R	N/R	N/R	N/R	**0.992**	**0.989**	**0.996**	**0.992**	N/R	**0.985**	N/R
Kumar and Kumar [15]	N/R	N/R	N/R	N/R	N/R	0.87	0.69	0.79	0.79	0.6	N/R
Rajpurkar et al. [6]	N/R	N/R	N/R	N/R	N/R	N/R	0.815	N/R	**0.929**	0.729	N/R

N/R = not reported by the cited study. TN/FP/FN/TP not reported by prior studies. Alzubaidi et al. [12]: shoulder-specific model, best fusion configuration. Kumar and Kumar [15]: shoulder results from second-stage classifier. Rajpurkar et al. [6]: DenseNet-169 baseline; AUC = AUROC; kappa from reported model.

**Table 10:** Validation, test, and prior study comparison for the elbow fracture dataset. Bold values indicate the best result in each metric column. Upward arrows (↑) indicate that higher values are better, while downward arrows (↓) indicate that lower values are better. N/R = not reported by the cited study.

Split	TN	FP	FN	TP	Accuracy ↑	Precision ↑	Recall ↑	F1-Score ↑	AUC ↑	Cohen’s Kappa ↑	ECE ↓
Validation	513	72	87	315	0.8389	0.8383	**0.8389**	0.8384	0.9011	0.6644	0.5224
Test	216	19	49	181	0.8538	**0.9050**	0.7870	0.8419	**0.9080**	0.7071	**0.4300**
Prior State-of-the-Art Studies (MURA dataset)											
Kumar and Kumar [15]	N/R	N/R	N/R	N/R	**0.88**	0.89	0.8	**0.87**	0.87	**0.75**	N/R
Lysdahlgaard [14]	N/R	N/R	N/R	N/R	0.73	N/R	N/R	N/R	N/R	N/R	N/R
Htun and Tun [13]	N/R	N/R	N/R	N/R	0.721	N/R	0.651	N/R	N/R	N/R	N/R

N/R = not reported. Kumar and Kumar [15]: elbow results from first-stage classifier. Lysdahlgaard [14]: best test accuracy = 0.73 (DenseNet169) on MURA elbow subset. Htun and Tun [13]: results on fuzzy-enhanced MURA elbow images. Rajpurkar et al. [6]: kappa from reported model.

**Table 11:** Validation, test, and prior study comparison for the finger fracture dataset. Bold values indicate the best result in each metric column. Upward arrows (↑) indicate that higher values are better, while downward arrows (↓) indicate that lower values are better. N/R = not reported by the cited study.

Split	TN	FP	FN	TP	Accuracy ↑	Precision ↑	Recall ↑	F1-Score ↑	AUC ↑	Cohen’s Kappa ↑	ECE ↓
Validation	557	71	130	264	**0.8033**	0.7884	**0.6701**	**0.7244**	**0.9011**	0.5730	0.5224
Test	176	38	87	160	0.7289	**0.8081**	0.6478	0.7191	0.8300	0.4631	**0.4000**
Prior State-of-the-Art Studies (MURA dataset)											
Kumar and Kumar [15]	N/R	N/R	N/R	N/R	N/R	N/R	N/R	N/R	N/R	**0.6**	N/R
Rajpurkar et al. [6]	N/R	N/R	N/R	N/R	N/R	N/R	N/R	N/R	N/R	0.389	N/R

N/R = not reported. No prior study reports per-metric finger-specific accuracy, precision, recall, F1, or AUC on MURA finger independently. Kappa values from Rajpurkar et al. [6] and Kumar and Kumar [15].

**Table 12:** Validation, test, and prior study comparison for the forearm fracture dataset. Bold values indicate the best result in each metric column. Upward arrows (↑) indicate that higher values are better, while downward arrows (↓) indicate that lower values are better. N/R = not reported by the cited study.

Split	TN	FP	FN	TP	Accuracy ↑	Precision ↑	Recall ↑	F1-Score ↑	AUC ↑	Cohen’s Kappa ↑	ECE ↓
Validation	211	22	44	88	0.8192	0.8000	0.6667	0.7273	0.89	0.5937	0.58
Test	144	6	51	100	0.8106	0.9434	0.6623	0.7782	0.88	0.6216	**0.45**
Prior State-of-the-Art Studies (MURA dataset)											
Kumar and Kumar [15]	N/R	N/R	N/R	N/R	**0.82**	0.93	**0.69**	**0.79**	0.82	0.64	N/R
Rajpurkar et al. [6]	N/R	N/R	N/R	N/R	N/R	N/R	N/R	N/R	N/R	**0.737**	N/R

N/R = not reported. Kumar and Kumar [15]: forearm results from second-stage classifier. Rajpurkar et al. [6]: kappa from reported DenseNet-169 model. Bold = best value per column.

**Table 13:** Validation, test, and prior study comparison for the hand fracture dataset. Bold values indicate the best result in each metric column. Upward arrows (↑) indicate that higher values are better, while downward arrows (↓) indicate that lower values are better. N/R = not reported by the cited study.

Split	TN	FP	FN	TP	Accuracy ↑	Precision ↑	Recall ↑	F1-Score ↑	AUC ↑	Cohen’s Kappa ↑	ECE ↓
Validation	629	183	98	199	0.7466	0.7730	0.7466	0.7555	0.7829	0.4077	0.6302
Test	257	14	101	88	0.7500	0.7774	**0.7500**	0.7299	0.7693	0.4449	**0.5066**
Prior State-of-the-Art Studies (MURA dataset)											
Kumar and Kumar [15]	N/R	N/R	N/R	N/R	**0.80**	**0.87**	0.69	**0.76**	**0.79**	0.60	N/R
Rajpurkar et al. [6]	N/R	N/R	N/R	N/R	N/R	N/R	N/R	N/R	N/R	**0.851**	N/R

N/R = not reported. Kumar and Kumar [15]: hand results from second-stage classifier average. Rajpurkar et al. [6]: kappa from reported DenseNet-169 model.

**Table 14:** Validation, test, and prior study comparison for the humerus fracture dataset. Bold values indicate the best result in each metric column. Upward arrows (↑) indicate that higher values are better, while downward arrows (↓) indicate that lower values are better. N/R = not reported by the cited study.

Split	TN	FP	FN	TP	Accuracy ↑	Precision ↑	Recall ↑	F1-Score ↑	AUC ↑	Cohen’s Kappa ↑	ECE ↓
Validation	120	15	19	101	0.8667	0.8668	0.8667	0.8665	0.9293	0.7319	0.5060
Test	125	23	21	119	0.8472	0.8473	0.8472	0.8472	0.9000	0.6943	**0.4588**
Prior State-of-the-Art Studies (MURA dataset)											
Kumar and Kumar [15]	N/R	N/R	N/R	N/R	**0.87**	**0.89**	**0.87**	**0.87**	0.87	**0.74**	N/R
Kutbi et al. [4]	N/R	N/R	N/R	N/R	0.8	N/R	N/R	N/R	N/R	N/R	N/R
Rajpurkar et al. [6]	N/R	N/R	N/R	N/R	N/R	N/R	N/R	N/R	N/R	0.6	N/R

N/R = not reported. Kumar and Kumar [15]: humerus from first-stage classifier. Kutbi et al. [4]: in-domain accuracy = 0.800 (ShuffleNetV2). Rajpurkar et al. [6]: kappa from reported model.

**Table 15:** Validation, test, and prior study comparison for the wrist fracture dataset. Bold values indicate the best result in each metric column. Upward arrows (↑) indicate that higher values are better, while downward arrows (↓) indicate that lower values are better. N/R = not reported by the cited study.

Split	TN	FP	FN	TP	Accuracy ↑	Precision ↑	Recall ↑	F1-Score ↑	AUC ↑	Cohen’s Kappa ↑	ECE ↓
Validation	1049	105	207	591	0.8402	0.8409	0.8402	0.8381	0.8935	0.6627	0.5597
Test	340	24	73	222	**0.8528**	**0.8587**	**0.8528**	**0.8508**	0.8771	0.6976	**0.5246**
Prior State-of-the-Art Studies (MURA dataset)											
Kutbi et al. [4]	N/R	N/R	N/R	N/R	0.848	N/R	N/R	N/R	N/R	N/R	N/R
Lysdahlgaard [14]	N/R	N/R	N/R	N/R	0.84	N/R	N/R	N/R	N/R	N/R	N/R
Rajpurkar et al. [6]	N/R	N/R	N/R	N/R	N/R	N/R	N/R	N/R	N/R	**0.931**	N/R

N/R = not reported. Kutbi et al. [4]: in-domain wrist accuracy = 0.8481 (ShuffleNetV2). Lysdahlgaard [14]: best test accuracy = 0.84 (VGG16) on MURA wrist. Rajpurkar et al. [6]: kappa from reported DenseNet-169 model.

**Table 16:** Comparison of Cohen’s kappa between radiologists, reported reference model, and the proposed Xception-Swin model.

Body Part	Radiologist 1	Radiologist 2	Radiologist 3	Reported Model	Proposed (Test)
Elbow	0.850 (0.830, 0.871)	0.710 (0.674, 0.745)	0.719 (0.685, 0.752)	0.710 (0.674, 0.745)	0.7071
Finger	0.304 (0.249, 0.358)	0.403 (0.339, 0.467)	0.410 (0.358, 0.463)	0.389 (0.332, 0.446)	0.4631
Forearm	0.796 (0.772, 0.821)	0.802 (0.779, 0.825)	0.798 (0.774, 0.822)	0.737 (0.707, 0.766)	0.6216
Hand	0.661 (0.623, 0.698)	0.927 (0.917, 0.937)	0.789 (0.762, 0.815)	0.851 (0.830, 0.871)	0.4449
Humerus	0.867 (0.850, 0.883)	0.733 (0.703, 0.764)	0.933 (0.925, 0.942)	0.600 (0.558, 0.642)	0.6943
Shoulder	0.864 (0.847, 0.881)	0.791 (0.765, 0.816)	0.864 (0.847, 0.881)	0.729 (0.697, 0.760)	0.6159
Wrist	0.791 (0.766, 0.817)	0.931 (0.922, 0.940)	0.931 (0.922, 0.940)	0.931 (0.922, 0.940)	0.6976

**Table 17:** Component-level ablation results for hand and humerus X-ray classification using Xception, Swin-Tiny, and the proposed Hybrid Attention model.

Body Part	Model	Component	Fusion	Best Epoch	Val. Accuracy	Val. Macro F1	Test Accuracy	Test Macro F1
Hand	Xception	CNN	None	9	73.95%	64.51%	66.30%	62.23%
	Swin-Tiny	Transformer	None	30	80.81%	73.07%	75.22%	72.36%
	Hybrid Attention	CNN + Transformer	Attention	5	82.87%	81.22%	75.00%	72.99%
Humerus	Xception	CNN	None	21	71.58%	71.08%	76.74%	76.53%
	Swin-Tiny	Transformer	None	27	79.21%	78.97%	80.90%	80.88%
	Hybrid Attention	CNN + Transformer	Attention	11	83.92%	83.92%	84.72%	84.72%

**Table 18:** Comparative summary of previous studies and the proposed framework based on performance measures and explainable AI.

Study	Dataset/Scope	Best Reported Performance	Cohen’s Kappa	Explainable AI
Rajpurkar et al. [6]	MURA, upper-extremity abnormality detection	AUROC = 0.9290, sensitivity = 0.8150, specificity = 0.8870	Overall = 0.7050	Yes; CAM
Majid et al. [11]	MURA, body-part + abnormality classification	Body-part accuracy = 0.9210; abnormality average accuracy = 0.7329; peak accuracy = 0.8162; macro F1 = 0.6880	Not reported	No
Kutbi et al. [4]	MURA + FracAtlas, zero-shot cross-part abnormality detection	In-domain accuracy up to 0.8481 (wrist), 0.8038 (elbow), 0.8000 (humerus); cross-domain accuracy up to 0.7630	Not reported	Yes; Grad-CAM
Alzubaidi et al. [12]	MURA, shoulder abnormality detection	Accuracy = 0.9920, specificity = 0.9890, recall = 0.9960, precision = 0.9890, F1 = 0.9920	0.9850	Yes; Grad-CAM, activation visualization, LIME
Htun and Tun [13]	MURA, elbow fracture/non-fracture classification	Accuracy = 0.7210, recall = 0.6513 on fuzzy-enhanced images	Not reported	No
Lysdahlgaard [14]	MURA, wrist and elbow abnormality detection	Wrist average accuracy = 0.8100, best = 0.8400; elbow average accuracy = 0.6000, best = 0.7300	Not reported	Yes; Grad-CAM
Kumar and Kumar [15]	MURA, two-stage body-part + abnormality diagnosis	Stage-1 F1 = 0.9700; Stage-2 accuracy up to 0.8800, AUC = 0.8700, F1 = 0.8700	Up to 0.7500	No
Singh et al. [16]	MURA + LERA, upper- and lower-extremity abnormality detection	Study-type classification accuracy = 97.37%; abnormality prediction accuracy = 89.00%; ROC-AUC = 0.94; sensitivity = 0.86; specificity = 0.89	0.77	No
Malau and Olusanya [17]	MURA; wrist anomaly detection with external cross-domain evaluation	Hybrid CNN-ViT wrist anomaly detection using parallel and sequential fusion with multistage transfer learning	Parallel Xception-DeiT: accuracy = 0.880 (internal MURA wrist); sequential DenseNet-ViT: recall = 0.900, AUC = 0.850 (external cross-domain)	Not reported
Proposed Xception-Swin	MURA, body-part classification + body-part-wise abnormality detection	Body-part classification: accuracy = 0.9643, macro F1 = 0.9574, AUC = 0.9963; abnormality detection examples: shoulder test accuracy = 0.8082, elbow = 0.8538, humerus = 0.8472, wrist = 0.8528	Body-part classification = 0.9579; test kappa up to 0.7071 (elbow), 0.6976 (wrist), 0.6943 (humerus)	Yes; Grad-CAM, Grad-CAM++, occlusion sensitivity

## Data Availability

The data presented in this study are openly available in MURA: Large Dataset for Abnormality Detection in Musculoskeletal Radiographs at https://doi.org/10.48550/arXiv.1712.06957 (accessed on 28 June 2025). The implementation resources are available at Zenodo: https://doi.org/10.5281/zenodo.19170555 (accessed on 28 June 2025).

## Supplementary Materials

The following supporting information can be downloaded at: https://www.mdpi.com/article/10.3390/jimaging12070298/s1, Algorithm S1: Pseudocode for the complete two-stage Xception-Swin framework. Phases A through F correspond to components 1 through 7 of Figure 1 in the main manuscript. Keywords are shown in bold, and comments are shown in italic grey; Table S1: FracAtlas external holdout dataset composition used for zero-shot cross-dataset evaluation; Table S2: Overall binary fracture/non-fracture classification performance on the full FracAtlas dataset (4083 images) using zero-shot transfer from the MURA-trained Xception-Swin model. ECE is reported in the lower-is-better direction; Table S3: Region-specific binary fracture/non-fracture classification results on FracAtlas using anatomically mapped MURA-trained Xception-Swin models. Direct indicates an exact anatomical region match, and indirect indicates the nearest anatomical analogue. Bold values indicate the best result per column across regions; Table S4: Comparison of within-domain MURA test accuracy and AUC versus cross-domain FracAtlas transfer accuracy and AUC for all four mapped MURA body-part models. Direct indicates an exact anatomical match, and indirect indicates the nearest anatomical analogue.

## Abbreviations

The following abbreviations are used in this manuscript:

AI	Artificial Intelligence
AMP	Automatic Mixed Precision
AUC	Area Under the Curve
AUC-ROC	Area Under the Receiver Operating Characteristic Curve
CAM	Class Activation Map
CLAHE	Contrast Limited Adaptive Histogram Equalization
CNN	Convolutional Neural Network
DL	Deep Learning
DSC	Dice Similarity Coefficient
ECE	Expected Calibration Error
F1	F1-Score
Grad-CAM	Gradient-weighted Class Activation Mapping
MURA	Musculoskeletal Radiographs
ROC	Receiver Operating Characteristic
XAI	Explainable Artificial Intelligence
