# Supplementary material for: Explainable Two-Stage Xception-Swin Transformer Learning for Body-Part-Aware Fracture Detection in Musculoskeletal X-Rays
Source: J Imaging. 2026 Jul 3;12(7):298. doi: 10.3390/jimaging12070298 (PMC13412450; doi:10.3390/jimaging12070298)
Supplement: Supplementary file 1 [file jimaging-12-00298-s001.zip › Cleaned Supplementary Algorithm Pseudocode.pdf]

Supplementary Material

Explainable Two-Stage Xception-Swin Transformer Learning for Body-Part-Aware Fracture Detection in Musculoskeletal X-Rays

Syed Baqir Hussain Shah [1], Musfarah Wajid [1], Syed Adil Hussain Shah [2,3], Silvia Godio [3], Karim Kassem [3,4], Gohar Bano Zaidi [3], Shahzad Ahmad Qureshi [5], Syed Taimoor Hussain Shah [3,*] and Marco Agostino Deriu [3,*]

[1] Department of Computer Science, COMSATS University Islamabad (CUI), Wah Campus, Wah 47000, Pakistan; bakirhussain6@gmail.com (S.B.H.S.); musfarahwajid@gmail.com (M.W.)
[2] Department of Research and Development (R&D), GPI SpA, Trento 38123, Italy; syedadilhussain.shah@gpi.it
[3] PolitoBIOMed Lab, Department of Mechanical and Aerospace Engineering, Politecnico di Torino, Turin 10129, Italy; silvia.godio@polito.it (S.G.); karim.kassem@polito.it (K.K.); gohar.zaidi@polito.it (G.B.Z.)
[4] Centro Medico Santagostino, Milan 20127, Italy
[5] Department of Computer and Information Sciences, Pakistan Institute of Engineering and Applied Sciences (PIEAS), Islamabad 45650, Pakistan; drsaqureshi@pieas.edu.pk
* Correspondence: taimoor.shah@polito.it (S.T.H.S.); marco.deriu@polito.it (M.A.D.); Tel.: +39-3517984023 (S.T.H.S.)

Algorithm S1. Pseudocode for the complete two-stage Xception-Swin framework. Phases A through F correspond to components 1 through 7 of Figure 1 (main manuscript). Keywords are shown in bold. Comments are shown in italic grey.

**Two-Stage Xception-Swin Framework for Musculoskeletal Radiograph Analysis**
**Input:** MURA dataset D (40,561 images, 7 body-part classes, binary abnormality labels)<br>**Output:** Body-part label and abnormality prediction with confidence score for each input radiograph X

**Phase A - Data Preparation (Figure 1, components 1 and 3)**
**for each** image I in D **do**<br>Apply CLAHE (clip limit = 2.0, tile grid = 8×8)<br>Apply fast non-local means denoising (h = 7, template = 7, search = 21)<br>Apply unsharp masking (amount = 0.8, radius = 1.2, threshold = 3)<br>Resize to 224×224; normalize with ImageNet mean [0.485, 0.456, 0.406] and std [0.229, 0.224, 0.225]<br>**end for**<br>Partition D into D_train (70%), D_val (30%), D_test (independent) // *stratified by class label*<br>Apply online augmentation to D_train only // *Crop, Flip, Rotation, Jitter, Gaussian noise, RandomErasing*
**Phase B - Architecture Screening: Body-Part Classification (Figure 1, component 2)**

for each model M in {DenseNet-201, ResNet-101, VGG-19, InceptionV3, EfficientNet-B0, Xception-Swin} **do**
Initialize M with ImageNet-pretrained weights
Fine-tune M on D_train for 7-class body-part classification (AdamW, CosineAnnealingLR)
Evaluate M on D_test; record Accuracy, F1-macro, AUC-ROC, Cohen's kappa
end for
Select M* = argmax(kappa) across all M *// Xception-Swin selected: accuracy = 0.9643, kappa = 0.9579*

Phase C - Hybrid Model Construction (Figure 1, component 4)

Load Xception backbone (ImageNet weights); apply global average pooling to obtain F_x
Load Swin-Tiny backbone (Patch4, Window7, ImageNet weights); apply global average pooling to obtain F_s
Fusion Path 1 (attention-based late fusion):
Project F_x and F_s to 512-dimensional latent space
Apply MultiheadAttention (8 heads, dropout = 0.1) over projected token pair [p_x, p_s]
Pass fused representation through Linear-GELU-Dropout(0.3)-Linear classification head (hidden = 256)
Fusion Path 2 (multi-scale spatial fusion):
Extract multi-scale feature maps from Swin-Tiny (out_indices = 0,1,2,3) and Xception (out_indices = 2,3)
Project via 1×1 convolutions to 128 channels; spatially align and concatenate
Apply Conv-BN-ReLU fusion blocks; global average pooling; Dropout(0.4); FC classification layer

Phase D - Multi-Phase Training (Figure 1, component 5)

Phase 1 (epochs 0-20%): Freeze both backbones; train fusion layer and classifier head only
Phase 2 (epochs 20-50%): Unfreeze final Xception blocks and last Swin stage; lr_backbone = 1e-5, lr_head = 1e-4
Phase 3 (epochs 50-100%): Unfreeze full model; lr_backbone = 1e-5, lr_head = 1e-4, weight_decay = 1e-4
Optimize with AdamW; CosineAnnealingLR (eta_min = 1e-7); label smoothing = 0.1; AMP enabled
Apply WeightedRandomSampler (inverse-frequency weights) to address class imbalance
Early stopping on D_val (patience = 12, min_delta = 1e-4); save best checkpoint M*_best by AUC

Phase E - Two-Stage Inference (Figure 1, component 6)

Stage 1: Given input radiograph X:
Predict body-part label b = M*_best(X) *// b in {elbow, finger, forearm, hand, humerus, shoulder, wrist}*
Stage 2: Load body-part-specific model M_b fine-tuned on subset D_b:
Predict abnormality label y = M_b(X) *// y in {normal, abnormal} with confidence score p*
return (b, y, p)

Phase F - Evaluation and Explainability (Figure 1, component 7)

for each body part b in {elbow, finger, forearm, hand, humerus, shoulder, wrist} **do**
Evaluate M_b on D_test; compute Accuracy, Precision, Recall, F1, AUC, Cohen's kappa, ECE (15 bins)
end for
for each test sample X in D_test **do**
Generate Grad-CAM map: backpropagate class gradients to last convolutional feature layer
Generate Grad-CAM++ map: weighted gradient aggregation over last convolutional feature layer
Generate Occlusion Sensitivity map: iteratively mask patches; measure confidence drop
Overlay heatmaps on X; confirm model attention on clinically relevant osseous regions
end for
