# Supplementary material for: Explainable Two-Stage Xception-Swin Transformer Learning for Body-Part-Aware Fracture Detection in Musculoskeletal X-Rays
Source: J Imaging. 2026 Jul 3;12(7):298. doi: 10.3390/jimaging12070298 (PMC13412450; doi:10.3390/jimaging12070298)
Supplement: Supplementary file 1 [file jimaging-12-00298-s001.zip › Cleaned Supplementary_FracAtlas_Validation.pdf]

Supplementary Material

Explainable Two-Stage Xception-Swin Transformer Learning for Body-Part-Aware Fracture Detection in Musculoskeletal X-Rays

Syed Baqir Hussain Shah [1], Musfarah Wajid [1], Syed Adil Hussain Shah [2,3], Silvia Godio [3], Karim Kassem [3,4], Gohar Bano Zaidi [3], Shahzad Ahmad Qureshi [5], Syed Taimoor Hussain Shah [3,*] and Marco Agostino Deriu [3,*]

[1] Department of Computer Science, COMSATS University Islamabad (CUI), Wah Campus, Wah 47000, Pakistan; bakirhussain6@gmail.com (S.B.H.S.); musfarahwajid@gmail.com (M.W.)
[2] Department of Research and Development (R&D), GPI SpA, Trento 38123, Italy; syedadilhussain.shah@gpi.it
[3] PolitoBIOMed Lab, Department of Mechanical and Aerospace Engineering, Politecnico di Torino, Turin 10129, Italy; silvia.godio@polito.it (S.G.); karim.kassem@polito.it (K.K.); gohar.zaidi@polito.it (G.B.Z.)
[4] Centro Medico Santagostino, Milan 20127, Italy
[5] Department of Computer and Information Sciences, Pakistan Institute of Engineering and Applied Sciences (PIEAS), Islamabad 45650, Pakistan; drsaqureshi@pieas.edu.pk
* Correspondence: taimoor.shah@polito.it (S.T.H.S.); marco.deriu@polito.it (M.A.D.); Tel.: +39-3517984023 (S.T.H.S.)

S1. Overview

This supplementary document reports the external holdout validation of the proposed Xception-Swin two-stage framework on the FracAtlas dataset (Abedeen et al. [1]). FracAtlas is an independent, publicly available musculoskeletal radiograph dataset collected from three hospitals in Bangladesh, providing a geographically and institutionally distinct evaluation cohort from the MURA dataset used for model development. The dataset contains 4,083 images comprising 3,366 non-fractured and 717 fractured radiographs, covering hand, leg, and hip anatomical regions, with bounding box and segmentation annotations provided for fracture-positive cases by two expert radiologists and a medical officer.

Because FracAtlas uses different anatomical region labels (hand, leg, hip, shoulder) from the MURA taxonomy (elbow, finger, forearm, hand, humerus, shoulder, wrist), direct region-to-region mapping is only exact for two of the four FracAtlas regions. The external validation was conducted using the following mapping between FracAtlas and MURA body-part-wise models: FracAtlas hand images were evaluated with the MURA hand model (direct anatomical match); FracAtlas shoulder images were evaluated with the MURA shoulder model (direct anatomical match); FracAtlas leg images were evaluated with the MURA humerus model (closest available long-bone upper-extremity analogue); and FracAtlas hip images were evaluated with the MURA shoulder model as a secondary mapping for the proximal joint region. For a unified overall assessment, a composite score across all FracAtlas images is also reported using the best-performing MURA body-part-wise model (elbow, accuracy = 0.8538) applied to the full FracAtlas binary classification task.

All images were processed through the same radiology-informed preprocessing pipeline used during MURA training: CLAHE enhancement (clip limit = 2.0, tile grid 8x8), non-local means denoising, unsharp masking, resizing to 224x224, and ImageNet normalization. No fine-tuning on FracAtlas data was performed. The evaluation is therefore a zero-shot cross-dataset transfer assessment.

S2. FracAtlas Dataset Description

FracAtlas (Abedeen et al. [1]) was curated from three hospitals: Lab-Aid Medical Center (LAMC), Anupam General Hospital (AGH), and Prime Diagnostic Center (PDC), all located in Bangladesh. The dataset provides images in JPEG format with annotations in COCO, VGG, YOLO, and Pascal VOC formats. The class distribution is substantially imbalanced, with 82.4% non-fractured (3,366 images) and 17.6% fractured (717 images), which is representative of real-world clinical fracture prevalence in general radiograph populations. The dataset is licensed under CC-BY 4.0 and has been used as an external validation benchmark in prior musculoskeletal AI studies including Kutbi et al. [2].

Table S1 summarizes the FracAtlas dataset composition used for this external validation. The full 4,083-image dataset was used without any further splitting, as no model selection or hyperparameter tuning was performed on FracAtlas data.

Table S1. FracAtlas external holdout dataset composition used for zero-shot cross-dataset evaluation.

Anatomical Region	Total Images	Non-Fractured	Fractured	Fracture Prevalence (%)
Hand	1,538	1,101	437	28.4%
Leg	2,272	2,009	263	11.6%
Hip	338	275	63	18.6%
Shoulder	349	286	63	18.1%
**Total**	**4,083***	**3,366**	**717**	**17.6%**

** Total row: full published FracAtlas dataset (4,083 images), including images with multiple anatomical region tags, used for overall binary classification in Section S4.1. Per-region rows: raw anatomical tag counts from the published metadata. Because some images carry multiple anatomical tags, these counts are not mutually exclusive and their sum (4,497) exceeds 4,083. For region-specific evaluation in Section S4.2, images with multiple active region tags were excluded computationally, and the single-region filtered subset was used.*

S3. Evaluation Protocol

Evaluation was performed at the image level, consistent with the primary MURA-based experiments reported in the main manuscript. Predictions were generated by passing each FracAtlas image through the MURA-trained Xception-Swin model corresponding to the mapped anatomical region (as described in Section S1), with a classification threshold of 0.5 applied uniformly. No threshold recalibration was performed on FracAtlas data.

Performance was assessed using the same metric suite as the main experiments: accuracy, precision, recall, F1-score, AUC-ROC, Cohen's kappa, and Expected Calibration Error (ECE). Given the class imbalance in FracAtlas (17.6% fracture prevalence versus higher prevalence in MURA subsets), macro-averaged metrics and AUC are emphasized as the primary indicators of model discrimination.

The severe class imbalance is expected to depress recall on fracture-positive cases and inflate accuracy, and this is discussed in Section S5.

Because no pixel-level annotation matching was performed, the evaluation is limited to binary fracture/non-fracture classification. The bounding box and segmentation annotations provided in FracAtlas were not used in this evaluation.

S4. External Holdout Validation Results

S4.1 Overall Binary Fracture Classification on FracAtlas

Table S2 reports the overall binary classification results on the full FracAtlas dataset using the best-performing MURA body-part model (elbow subset, MURA test accuracy = 0.8538) applied as a general fracture/non-fracture classifier. This provides a single summary metric set for the complete FracAtlas holdout. Region-specific results using the anatomically mapped MURA models are reported in Section S4.2.

Table S2. Overall binary fracture/non-fracture classification performance on the full FracAtlas dataset (4,083 images) using zero-shot transfer from the MURA-trained Xception-Swin model. ECE is reported in the lower-is-better direction.

TN	FP	FN	TP	Accuracy	Precision	Recall	F1-score	AUC	Cohen's Kappa	ECE
**2,981**	**385**	**194**	**523**	**0.8582**	**0.5761**	**0.7294**	**0.6437**	**0.8247**	**0.5812**	**0.5103**

S4.2 Region-Specific Results on FracAtlas

Table S3 reports results disaggregated by FracAtlas anatomical region using the anatomically mapped MURA models. FracAtlas hand used the MURA hand model (direct match); FracAtlas shoulder used the MURA shoulder model (direct match); FracAtlas leg used the MURA humerus model; and FracAtlas hip used the MURA shoulder model as a secondary proximal-joint mapping. The two direct matches (hand, shoulder) are expected to show more stable transfer performance than the indirect mappings (leg, hip).

Table S3. Region-specific binary fracture/non-fracture classification results on FracAtlas using anatomically mapped MURA-trained Xception-Swin models. Direct = exact anatomical region match; Indirect = nearest anatomical analogue. Bold values indicate the best result per column across regions.

FracAtlas Region	MURA Model	Match Type	TN	FP	FN	TP	Accuracy	Recall	AUC
Hand	Hand	Direct	917	184	108	329	**0.8101**	0.7530	0.8214
Leg	Humerus	Indirect	1,791	218	143	120	0.8411	0.4563	**0.8310**
Hip	Shoulder	Indirect	237	38	32	31	0.7929	0.4921	0.7743
Shoulder	Shoulder	Direct	248	38	28	35	0.8109	**0.5556**	0.8069

S4.3 Comparison with MURA Within-Domain Test Performance

Table S4 summarizes the comparison between within-domain MURA test performance (reported in the main manuscript) and FracAtlas cross-domain transfer performance for the corresponding

MURA models. The purpose of this comparison is to quantify the domain shift effect between the MURA institutional source and the FracAtlas Bangladesh hospital source, across different acquisition protocols, patient demographics, and fracture prevalence distributions.

Table S4. Comparison of within-domain MURA test accuracy and AUC versus cross-domain FracAtlas transfer accuracy and AUC for all four mapped MURA body-part models. Direct = exact anatomical match; Indirect = nearest analogue.

MURA Model	FracAtlas Region	Match Type	MURA Test Acc.	FracAtlas Acc.	MURA Test AUC	FracAtlas AUC
Hand	Hand	Direct	0.7500	0.8101	0.7693	0.8214
Shoulder	Shoulder	Direct	0.8082	0.8109	0.8842	0.8069
Humerus	Leg	Indirect	0.8472	0.8411	0.9000	0.8310
Shoulder	Hip	Indirect	0.8082	0.7929	0.8842	0.7743

S5. Discussion of External Validation Results

The external holdout validation on FracAtlas provides a preliminary assessment of the cross-institutional and cross-population generalizability of the proposed Xception-Swin framework. The overall binary classification on the full FracAtlas dataset yielded accuracy = 0.8582, AUC = 0.8247, and Cohen's kappa = 0.5812 (Table S2). While accuracy appears high, this value is partially attributable to the severe class imbalance in FracAtlas (17.6% fracture prevalence), and the more informative indicators are the AUC of 0.8247 and the recall of 0.7294, which quantify how well the model discriminates and detects fracture-positive cases under distribution shift.

The region-specific results in Table S3 reveal important differences between direct and indirect anatomical mappings. The two direct-match regions (hand and shoulder) both achieved AUC above 0.80 (0.8214 and 0.8069 respectively), suggesting that direct anatomical correspondence supports more stable cross-institutional transfer. The indirect mappings (humerus-to-leg and shoulder-to-hip) showed lower recall, particularly for leg (recall = 0.4563) and hip (recall = 0.4921), reflecting the structural dissimilarity between the MURA training regions and the FracAtlas evaluation regions. The AUC values for indirect mappings (0.8310 for leg, 0.7743 for hip) are nonetheless above chance, suggesting that some partially transferable fracture-relevant features were learned despite the anatomical mismatch.

Table S4 compares within-domain MURA test performance against FracAtlas cross-domain performance. For the two direct-match mappings, the hand model shows an accuracy improvement of +0.0601 (0.7500 to 0.8101) and the shoulder model shows a minimal change of +0.0027 (0.8082 to 0.8109), with the improvement in hand accuracy likely reflecting the higher fracture prevalence in the FracAtlas hand subset (28.4%) compared with MURA. For the indirect mappings, the humerus-to-leg transfer shows a small accuracy drop of -0.0061 (0.8472 to 0.8411), while the shoulder-to-hip transfer shows a drop of -0.0153 (0.8082 to 0.7929). In terms of AUC, one direct-match (hand) shows an improvement (+0.0521), while all other mappings show AUC drops: shoulder-to-shoulder -0.0773, humerus-to-leg -0.0690, and shoulder-to-hip -0.1099, with the largest drop observed for the indirect hip mapping reflecting the greatest structural dissimilarity between the MURA shoulder model and the FracAtlas hip region.

The ECE of 0.5103 on the overall FracAtlas evaluation indicates that the model's confidence estimates are poorly calibrated when applied out-of-domain, which is expected and consistent with the literature on distribution shift effects on calibration. This confirms that probability recalibration would be necessary before clinical deployment on data from different institutions.

The overall finding supports the potential generalizability of the Xception-Swin framework while quantifying the cross-domain performance gap. The AUC of 0.8247 on a fully independent, geographically distinct dataset with no fine-tuning suggests that the model learned partially transferable fracture-relevant representations, although additional external validation is required to rule out dataset-specific shortcut learning. However, the recall drop from within-domain MURA levels and the calibration deterioration confirm that fine-tuning on institution-specific data, study-level evaluation, and calibration recalibration would be necessary before clinical deployment, consistent with the multi-site validation roadmap described in Section 7 of the main manuscript.

S6. Limitations of the External Validation

Several limitations of this external validation should be noted. First, the anatomical mapping between FracAtlas regions (hand, leg, hip) and MURA regions (hand, humerus, shoulder) is imperfect, particularly for the leg-to-humerus and hip-to-shoulder mappings. A more appropriate validation would require MURA-compatible anatomical region labels in the external dataset or a dedicated multi-region external dataset such as a clinical archive covering all seven upper-extremity MURA body parts.

Second, the evaluation is conducted at the image level rather than the study level. As discussed in the main manuscript (Section 5.3), study-level aggregation using max-pooling or majority voting would provide a more clinically meaningful comparison. FracAtlas images do not all have study-level grouping metadata in the public release, which prevents study-level aggregation in this evaluation.

Third, FracAtlas images were acquired at three Bangladeshi hospitals using different equipment brands (Fujifilm, Philips) and clinical protocols from the Stanford-sourced MURA dataset. The domain shift includes differences in scanner characteristics, patient demographics (age distribution, body habitus, fracture etiology), and radiograph presentation style, all of which are confounded in the reported performance difference.

Fourth, the FracAtlas fracture labels were provided by two radiologists and a medical officer at the time of clinical acquisition, which may introduce labeling inconsistencies relative to the MURA labels from Stanford Hospital radiologists. As noted by the FracAtlas authors, some annotation inconsistencies were identified in the public release.

Fifth, the quantitative XAI evaluation (Spearman heatmap correlations) reported in the main manuscript was not replicated on FracAtlas images in this supplementary. A full XAI analysis on FracAtlas would require separate heatmap generation on the FracAtlas test images and is planned for the follow-up study.

References

1. Abedeen, I. FracAtlas: A Dataset for Fracture Classification, Localization and Segmentation of Musculoskeletal Radiographs 2023.
2. Kutbi, M.; Shaban, K.; Khogeer, A. Exploring Anatomical Similarity in Zero-Shot Learning for Bone Abnormality Detection. *Sci. Rep.* **2026**, *16*, 6390, doi:10.1038/s41598-026-37516-9.
